# RCOR1 promotes myoblast differentiation and muscle regeneration

**DOI:** 10.1038/s41420-025-02568-9

**Published:** 2025-07-01

**Authors:** Martina Pauk, Fan Wang, Petri Rummukainen, H. G. Mauricio Ramm, Hanna Taipaleenmäki, Riku Kiviranta

**Affiliations:** 1 Institute of Biomedicine, University of Turku, Turku, Finland; 2 Institute of Musculoskeletal Medicine, LMU University Hospital, LMU Munich, Munich, Germany; 3 Musculoskeletal University Center Munich, LMU University Hospital, LMU Munich, Munich, Germany; 4 Department of Molecular Medicine and Surgery, Karolinska Institutet, Stockholm, Sweden; 5 Department of Endocrinology, Division of Medicine, University of Turku and Turku University Hospital, Turku, Finland

**Keywords:** Physiology, Skeletal muscle

## Abstract

RCOR proteins belong to a family of highly conserved transcription corepressors (RCOR1, RCOR2 and RCOR3) that regulate the activity of associated histone demethylase 1 (LSD1) and histone deacetylase 1/2 (HDAC 1/2) in chromatin-modifying complexes. Despite the described function of LSD1 in skeletal muscle differentiation and regeneration, the role of RCOR family in myogenesis remains unknown. We found that RCOR1 is highly expressed in proliferating myoblasts and activated satellite cells, but not in mature myofibers during postnatal skeletal muscle growth and regeneration. Silencing of RCOR1 impaired myoblast differentiation and fusion, as evidenced by reduced levels of myogenin and MyHC, key markers of myogenic commitment. Moreover, RCOR1 depletion impaired myoblast proliferation through upregulation of the cell cycle inhibitor P21. Although combined silencing of P21 and RCOR1 rescued the proliferation defect of RCOR1 deficiency alone, it failed to restore differentiation, suggesting that RCOR1 action on myoblast proliferation and differentiation is mediated via independent mechanisms. RCOR1 was found physically associated with LSD1 and myogenic regulatory factor MyoD and contributed to LSD1 stability in myoblasts via ubiquitination. Accordingly, the repressive effect of RCOR1 depletion on myogenic differentiation was rescued by LSD1 overexpression, indicating that RCOR1 exerts its function on myoblast differentiation primarily through LSD1. Consistently, in a mouse model of skeletal muscle injury, depletion of RCOR1, accompanied with reduction of LSD1, supressed satellite cell activation and differentiation which resulted in impaired muscle regeneration. Together, our findings indicate that RCOR1 acts in concert with LSD1 as a novel positive regulator of myogenesis and skeletal muscle regeneration.

## Introduction

Skeletal muscle is the most abundant and highly complex tissue of the vertebrate body. The process of muscle formation known as myogenesis, occurs during embryonic development, postnatal growth and regeneration of adult skeletal muscle upon injury. Skeletal muscle has remarkable regenerative capacity mainly due to its resident population of paired box 7 (Pax7) -expressing muscle stem cells called satellite cells (SCs) [1]. During adult myogenesis, SCs are activated and form mononucleated myoblasts that exit the cell cycle, subsequently fusing into multinucleated myotubes and ultimately form mature muscle fibres [1]. These processes are regulated by a complex molecular network, including several transcription factors, among which four myogenic regulatory factors (MRFs), myoblast determination protein 1 (MyoD), myogenic factor 5 (Myf5), muscle regulatory factor 4 (Mrf4) and myogenin, have a vital role in controlling muscle-specific gene expression [1, 2]. MyoD, Myf5 and Mrf4 act as early muscle determination factors [3–5], while myogenin, induced by the other three MRFs, regulates the later stages of myogenic differentiation and the myofiber maturation [6, 7]. MRFs also cooperate with other transcriptional regulators such as myocyte enhancer factor 2 (Mef2) to regulate and activate muscle specific target genes which are required for sarcomeric organization and contractility, such as myosin heavy chain (MyHC) [8].

The epigenetic mechanisms involved in the regulation of myogenesis are not fully understood. Several studies have demonstrated an essential role of lysine-specific histone demethylase 1 (LSD1) in skeletal muscle differentiation and regeneration. LSD1 acts as a corepressor by demethylating mono- and di-methylated lysine 4 and/or 9 residues of histone H3 (H3K4me1/2 and H3K9me1/2), leading to either transcriptional repression or activation, respectively [9–11]. Inhibition of LSD1 has been shown to impair skeletal myoblast differentiation in C2C12 cells [12–14]. A proposed mechanism of action suggests direct binding of LSD1 and MyoD to the myogenin promoter, leading to demethylation of the di-methylated form of H3K9 and consequent activation of myogenic genes [12]. In addition, LSD1 was shown to be necessary for the timely expression of MyoD through the regulation of the MyoD core enhancer during muscle differentiation [15]. Finally, LSD1 was identified as a key epigenetic regulator of muscle regeneration by repressing brown adipocyte differentiation of SCs and promoting myogenesis [16].

LSD1 is found in association with the transcriptional corepressor CoREST complex, whose primary components are LSD1, histone deacetylase 1 or 2 (HDAC1/2) and REST Corepressor (RCOR) 1, 2 or 3 scaffold protein [17]. Within this complex, the presence of RCOR protein is essential for the nucleosome recognition and histone tail binding leading to effective demethylation and deacetylation by LSD1 and HDAC1/2, respectively [9, 17]. There are three vertebrate RCOR proteins RCOR1 (CoREST1), RCOR2 (CoREST2) and RCOR3 (CoREST3), encoded by separate genes. Although all three RCORs share high sequence similarity and ability to interact with LSD1, they possess differential biochemical properties and distinct molecular actions. RCOR2 and RCOR3 display lower transcriptional repressive capacity than RCOR1, which could be due to structural variations and altered protein-protein interactions [18]. Accordingly, RCOR1 and to a lesser extent RCOR2 efficiently stimulate LSD1-mediated demethylation of nucleosomes, while this was not observed for the LSD1-RCOR3 complex in erythroid cells [9, 19–21]. This led us to hypothesize that RCOR1 may be the primary RCOR protein that acts in concert with LSD1 in myoblasts and is involved in regulation of the myogenic gene programme. RCOR1 has been studied in a wide variety of cell types, and shown to play important roles in mouse erythropoiesis [22], neuronal gene regulation [23, 24] and tumorigenesis [25] by chromatin remodelling. However, its potential role in myogenesis has not been investigated.

Here, we demonstrated that RCOR1 expression is linked to the progression of myogenic differentiation, postnatal growth and muscle regeneration upon injury. Furthermore, silencing of RCOR1 substantially impaired myoblast proliferation and differentiation by supressing the expression of cell cycle regulators and key myogenic factors, respectively. RCOR1 depletion in muscle reduced activation and differentiation of SCs, leading to an impaired muscle regeneration following injury. Collectively, we have identified for the first time the specific role of RCOR1 in skeletal muscle differentiation and regeneration.

## Results

### RCOR1 is dynamically expressed during myoblast differentiation and in vivo myogenesis

**Fig. 1:**
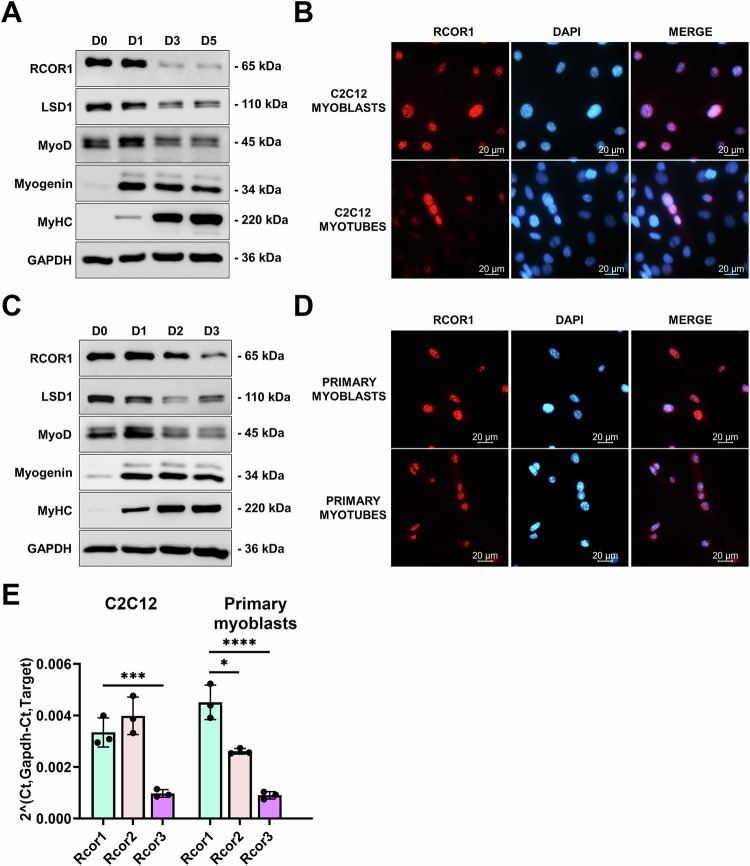
RCOR1 is dynamically expressed during myoblast differentiation. **A** C2C12 myoblasts cultured in growth medium (GM) were induced to differentiate to myotubes by switching to differentiation medium (DM) for 5 days. The expression of RCOR1, LSD1, MyoD, Myogenin and MyHC was detected by immunoblotting. GAPDH was used as loading control. **B** Immunofluorescence staining for RCOR1 was performed in C2C12 myoblasts and myotubes (red, RCOR1; blue, DAPI). Scale bars indicate 20 µm. **C** Primary mouse myoblasts cultured in GM were induced to differentiate into myotubes by switching to DM for 3 days. The expression levels of RCOR1, LSD1, MyoD, Myogenin and MyHC were detected by immunoblotting. GAPDH was used as loading control. **D** Immunofluorescence staining for RCOR1 was performed in primary mouse myoblasts and myotubes (red, RCOR1; blue, DAPI). Scale bars indicate 20 µm. **E** mRNA expression of *Rcor* family members *Rcor1, Rcor2 and Rcor3* was analysed by qPCR in C2C12 cells and primary myoblasts. Pictures and immunoblots show representatives of 3 independent experiments. Data are presented as mean ± SD; *P < 0.05; ***P < 0.001, ****P < 0.0001; One-way ANOVA was performed.

To determine whether RCOR1 is regulated during myogenesis, we analysed its expression and localization patterns in several myogenesis systems in vitro and in vivo. First, we used the mouse skeletal muscle cell line C2C12, which represents a well-established model of the skeletal muscle differentiation process. Differentiation was initiated upon serum deprivation leading C2C12 myoblasts to fuse into multinucleated myotubes during a 5-day period. *Rcor1* mRNA expression remained unchanged during this period (Fig S1A). However, immunoblotting showed that RCOR1 protein gradually declined throughout myoblast differentiation, along with an increase in the abundance of myogenic markers myogenin and MyHC (Fig. 1A). Interestingly, *Lsd1* mRNA expression moderately increased during differentiation, but protein abundance gradually decreased following the onset of myogenic differentiation (Figs. 1A and S1A), suggesting that both LSD1 and RCOR1 are reduced during myogenesis through post-transcriptional regulation. In order to analyse the cellular distribution of RCOR1, we performed immunofluorescence staining in C2C12 myoblasts. RCOR1 was mainly expressed in the nuclei of myoblasts and differentiated myotubes (Fig. 1B), which is in accordance with its role in chromatin modification. To confirm these findings in primary cells, we isolated primary myoblasts from male mice and induced them to differentiate to myotubes over a 3-day period. Consistently, both RCOR1 and LSD1 mRNA and protein decreased during the differentiation of primary myoblasts (Figs. 1C and S1B) and nuclear localization of RCOR1 was confirmed by immunostaining in both, myoblasts and myotubes (Fig. 1D). Furthermore, LSD1 was expressed in the nuclei of C2C12 cells (Fig. S1C). In addition, we investigated the expression levels of other members of RCOR family, *Rcor2* and *Rcor3*, during differentiation by qPCR. Similar to *Rcor1*, *Rcor2* and *Rcor3* mRNA expression remained unchanged during the C2C12 myoblast differentiation but decreased following the onset of primary myoblast differentiation (Fig S1D and E). Notably, *Rcor1* was expressed higher than *Rcor3* in C2C12 myoblasts and showed highest abundance in primary myoblasts compared to *Rcor2* and *Rcor3* (Fig. 1E).

**Fig. 2:**
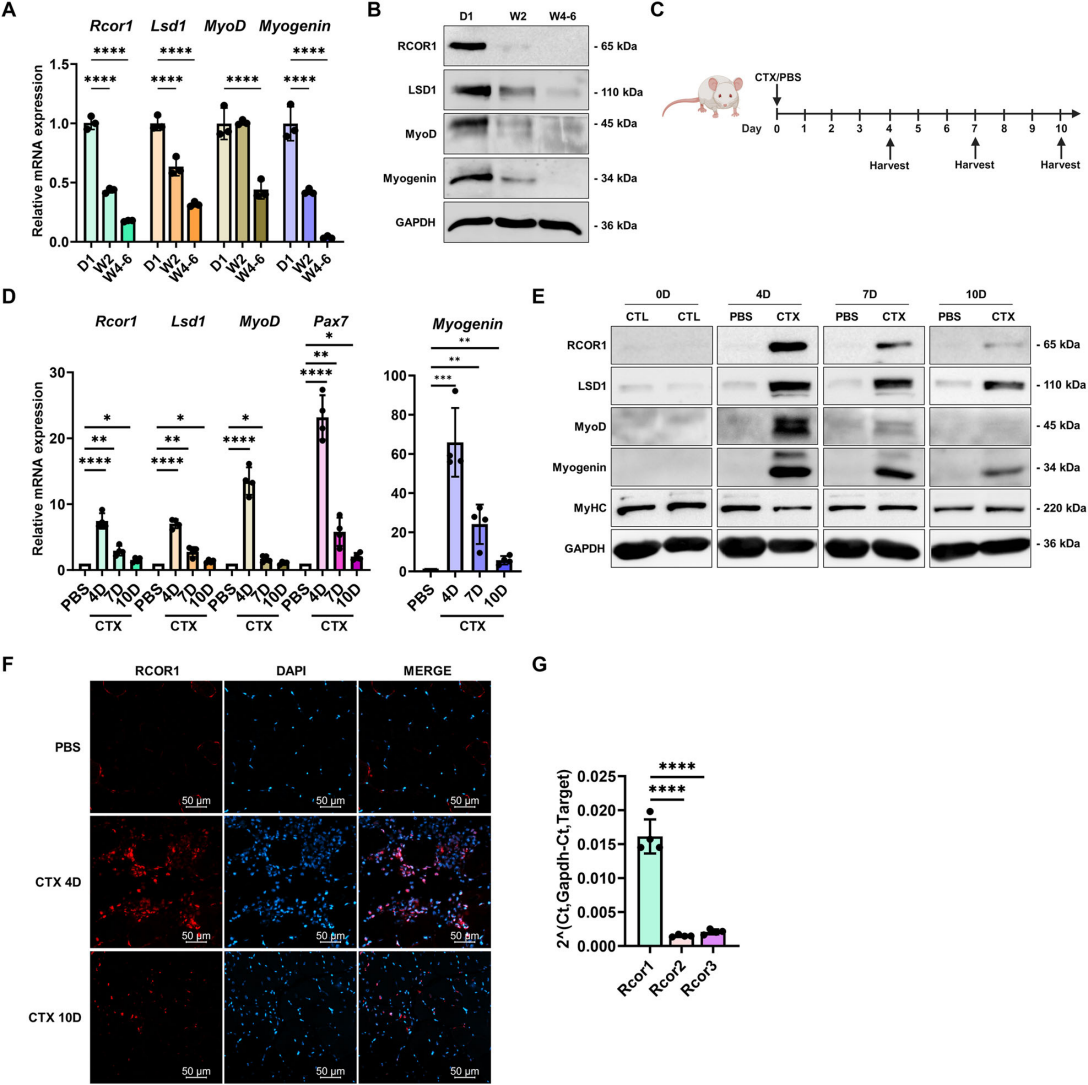
RCOR1 is dynamically expressed during in vivo myogenesis. **A** Hindlimb muscles from mice at postnatal day 1 (D1), week 2 (W2) and week 4–6 (W4–6) were analysed by qPCR and **B** immunoblotting to detect mRNA and protein abundance of RCOR1, LSD1 and myogenic regulatory factors MyoD and Myogenin. *n* = 3 mice per group. **C** Schematic representation of the injury model, in which TA muscles were injured with Cardiotoxin (CTX) or PBS injection and harvested on day 4, 7 and 10. **D** qPCR analysis of *Rcor1, Lsd1, MyoD, Pax7* and *Myogenin* mRNA expression. **E** Immunoblot analysis of RCOR1, LSD1, MyoD, Myogenin, and MyHC expression protein expression in the injured muscles. GAPDH was used as loading control. **F** Upon cryosection, immunofluorescence staining for RCOR1 (red) and DAPI (blue) in TA muscles was performed at day 4 and 10 after CTX injury. Scale bars indicate 50 µm. **G** mRNA expression of *Rcor* family members was analysed by qPCR at day 4 in CTX-injured TA muscle. *n* = 4 mice per group. Data are presented as mean ± SD; *P < 0.05; **P < 0.01; ***P < 0.001, ****P < 0.0001; One-way ANOVA was performed.

Next, we investigated the expression of RCOR1 during myogenesis in vivo. Both RCOR1 and LSD1 were highly expressed in limb muscles of newborn mice on postnatal day 1 (P1) but decreased in 2- and 4-week-old mice (Fig. 2A and B). Consistently, key myogenic markers MyoD and myogenin showed a similar expression pattern during muscle development with lower abundance in adult muscles, as expected (Fig. 2A and B). To explore the expression of RCOR1 during skeletal muscle regeneration, TA muscles were injured by CTX injection and allowed to regenerate for 10 days (Fig. 2C). Four days post injury, we observed extensive inflammatory cell infiltration accompanied by small regenerating myofibers with centrally located nuclei (Fig. S2). After 10 days of regeneration, the CTX-injured muscle appeared nearly fully regenerated with only few interstitial cells left. Both RCOR1 and LSD1 mRNA and protein expression were highly increased in CTX-treated TA muscle relative to contralateral PBS-treated muscle at 4 days following injection (Fig. 2D and E). At 7–10 days postinjury, RCOR1 and LSD1 expression gradually declined but remained detectable over the course of regeneration. RCOR1 was observed in mononucleated cells at the injury site and within newly formed muscle fibres in CTX-injured muscle (Fig. 2F). Importantly, satellite cell marker *Pax7* and myogenic markers, MyoD and myogenin, showed similar dynamics in the expression, consistent with their role in coordinating myogenesis (Fig. 2D and E). Consistent with previous findings in myoblasts, we found that *Rcor1* is the most abundant RCOR family member in CTX-injured muscle (Fig. 2G). Collectively, these data revealed that RCOR1 is associated with active myogenesis in vitro and in vivo and might play a role in skeletal muscle differentiation and regeneration.

### RCOR1 regulates myoblast differentiation

**Fig. 3:**
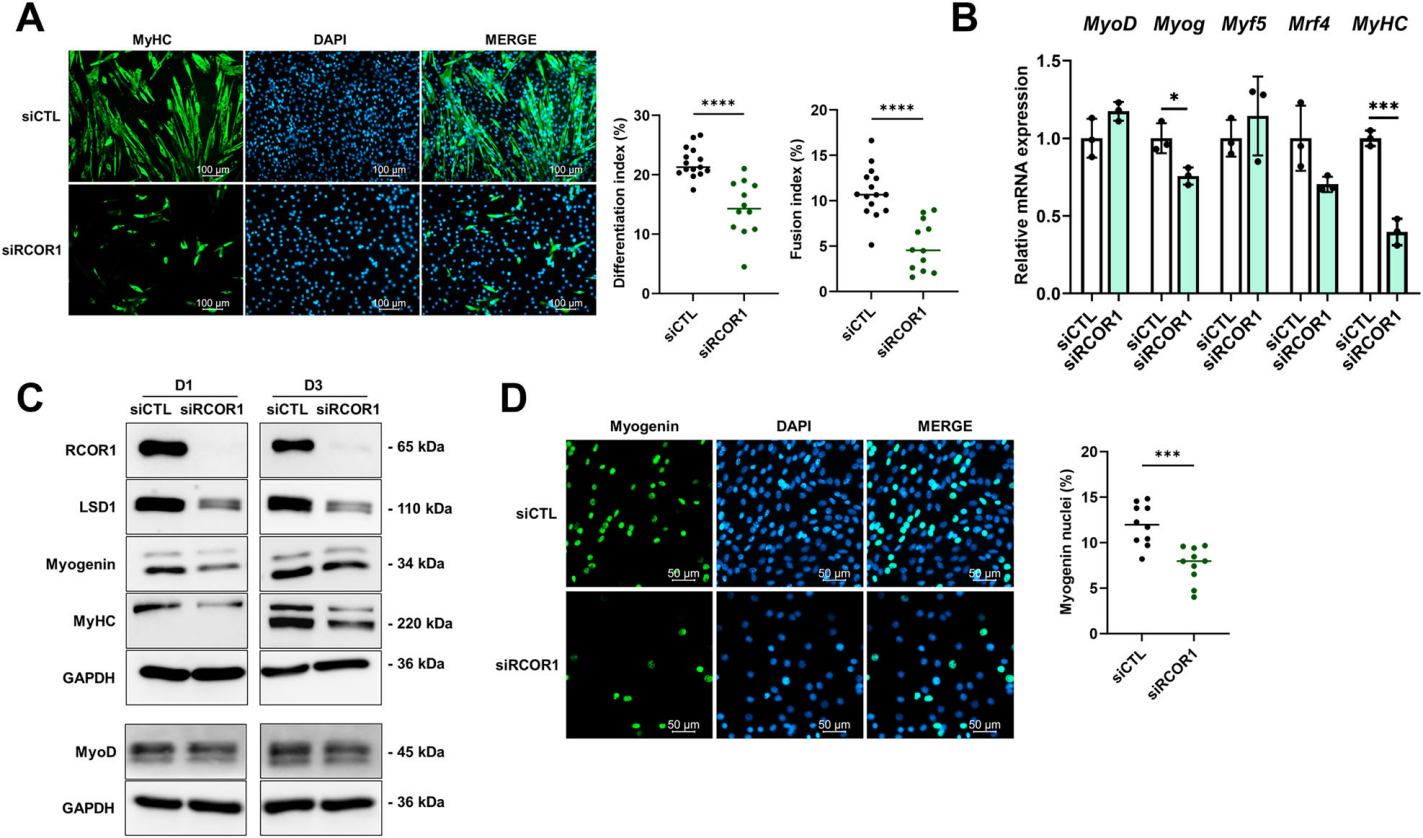
RCOR1 regulates myoblast differentiation. C2C12 cells transfected with control siRNA (siCTL) or RCOR1 siRNA (siRCOR1) were cultured in growth medium (GM) and induced to differentiate by switching to differentiation medium (DM) for 3 days. **A** Terminally differentiated myotubes were visualized by anti-MyHC immunofluorescent staining (green, MyHC; blue, DAPI) at day 3 in DM. The differentiation index and fusion index were analysed. Scale bars indicate 100 µm. **B** qPCR analysis of *MyoD, Myog, Myf5* and *Mrf4* mRNA expression at day 1 and *MyHC* at day 3 in DM. **C** Immunoblotting was performed to detect protein levels of RCOR1, LSD1, Myogenin and MyoD at day 1 and MyHC at day 3 in DM. GAPDH was used as loading control. **D** Immunofluorescence staining for Myogenin was performed at day 1 in DM (green, Myogenin; blue, DAPI) and analysed. Scale bars indicate 50 µm. Pictures show representatives of 3 independent experiments. Data are presented as means ± SD; *P < 0.05; ***P < 0.001, ****P < 0.0001; Student’s *t*-test was performed.

To investigate whether RCOR1 is required for skeletal myogenesis, we silenced RCOR1 in C2C12 myoblasts by using small interfering RNAs (siRNAs). Knockdown efficiency was verified by qPCR and immunoblotting (Fig. S3A). As shown by MyHC immunostaining, RCOR1-deficiency severely impaired myogenic differentiation and fusion as indicated by a lower differentiation and fusion index relative to control siRNA (Fig. 3A). siRCOR1-transfected myoblasts remained as individual mononucleated cells and did not fuse to form multinucleated myotubes. Consistently, the expression of myogenic regulatory factor myogenin and muscle structural protein MyHC was reduced at early and late stages of differentiation, respectively (Fig. 3B and C). Immunofluorescence staining of myogenin revealed a reduced number of myogenin positive myoblasts following RCOR1 silencing, supporting the inhibitory effect on the differentiation (Fig. 3D). Of note, the expression of myogenic regulatory factor MyoD remained unchanged during differentiation of C2C12 myoblasts (Fig. 3B and C). In primary cultures, siRCOR1 transfected myoblasts exhibited smaller and fewer myotubes relative to elongated multinucleated myotubes formed by control myoblasts (Fig. S3B). Consistent with the observed decrease in myotube formation, fusion index and mRNA expression of *myogenin*, *Mrf4* and *MyHC* were reduced in siRCOR1 transfected primary myoblasts compared to control (Fig. S3B and C). Both RCOR2 and RCOR3 associate with LSD1 and functional redundancy has been previously reported among the family members [18, 21, 26]. To determine whether other RCORs compensate for the loss of RCOR1, we examined the expression of *Rcor2* and *Rcor3* following RCOR1 silencing and found no changes in mRNA expression in C2C12 myoblasts (Fig. S3D).

**Fig. 4:**
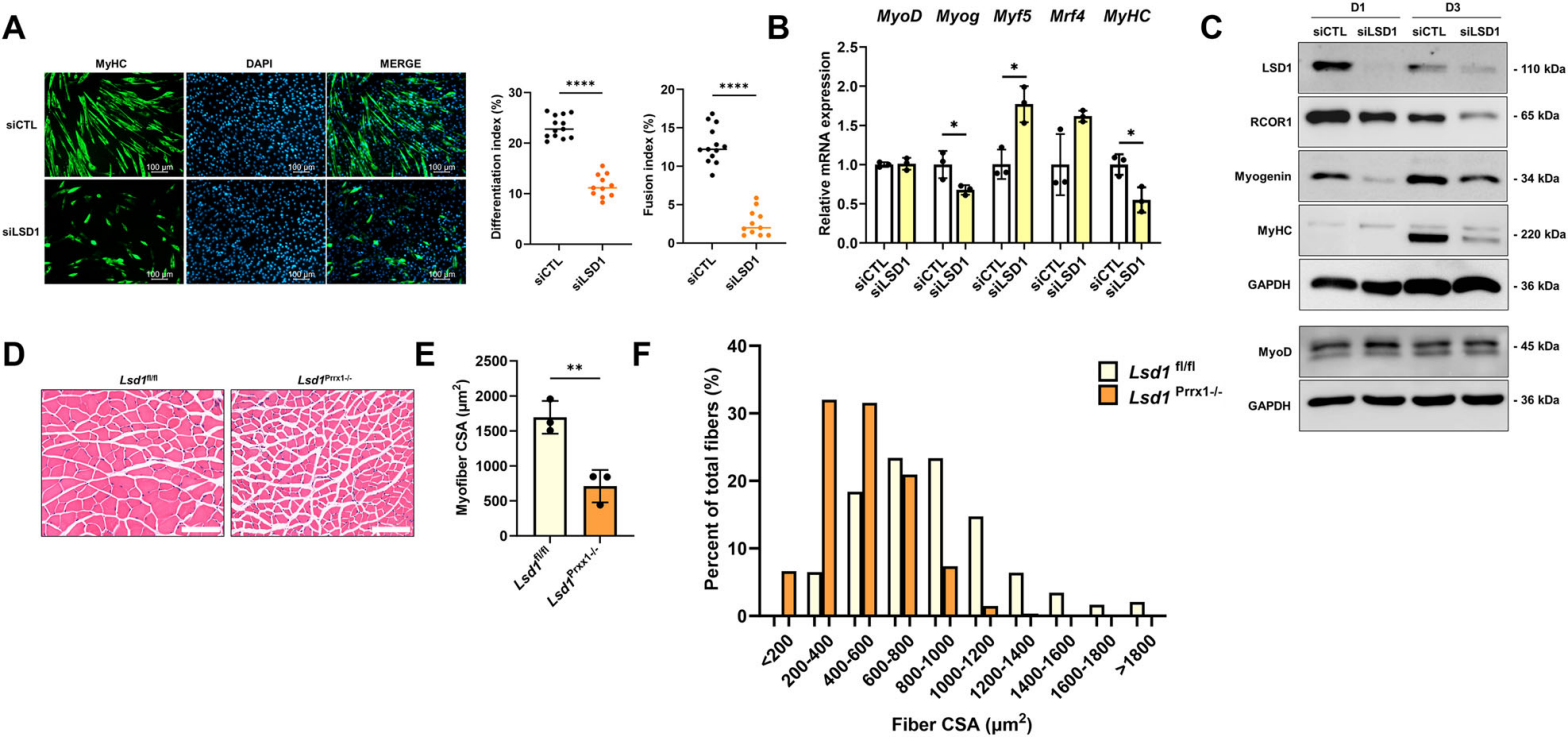
LSD1 deletion impairs myoblast differentiation and muscle development and growth. C2C12 cells transfected with control siRNA (siCTL) or LSD1 siRNA (siLSD1) were cultured in a growth medium (GM) and induced to differentiate by switching to differentiation medium (DM) for 3 days. **A** Terminally differentiated myotubes were visualized by anti-MyHC immunofluorescent staining (green, MyHC; blue, DAPI) on day 3 in DM. The differentiation index and fusion index were analysed. Scale bars indicate 100 µm. **B** qPCR analysis of *MyoD, Myog, Myf5*, *Mrf4* at day 1 and *MyHC* at day 3 in DM. **C** Immunoblotting was performed to detect protein abundance of RCOR1, LSD1, Myogenin and MyoD at day 1 and MyHC at day 3 in DM. GAPDH was used as a loading control. **D** TA muscles of 4 weeks old control (*Lsd1*^fl/fl^) and LSD1 knockout mice (*Lsd1*^Prrx1-/-^) were stained by hematoxylin and eosin (H&E) (Scale bar 100 µm) and analysed for **E** mean myofiber cross-sectional area (CSA) and **F** percent distributions of CSA (μm^2^). *n* = 3 mice per group. Pictures and blots are one representative of 3 independent experiments (**A**, **C**) or mice (**D**). Data are presented as means ± SD; *P < 0.05; **P < 0.01, ****P < 0.0001; Student’s *t*-test was performed.

As described above, RCOR1 is a part of the CoREST complex containing LSD1 and HDAC1/2 [9, 17, 27]. Moreover, RCOR1 directly binds to LSD1 and regulates its activity and function [18, 21]. Previous studies indicated that LSD1 plays a role in myogenic differentiation [12–14]. Similarly, we observed that silencing LSD1 impaired myoblast differentiation and fusion as shown by MyHC immunostaining in C2C12 cells and primary myoblasts (Figs. 4A and S4A). Furthermore, the loss of LSD1 reduced the expression of myogenin and MyHC (Figs. 4B and C and S4B). However, contrary to previous reports [12–14], LSD1 had no impact on MyoD mRNA and protein expression (Figs. 4B and C and S4B). Different functional approaches for LSD1 depletion and inactivation in mice have demonstrated the requirement of LSD1 in myogenic differentiation and muscle regeneration [15, 16, 28, 29]. Here, we crossed *Lsd1*^*fl/fl*^ mice with *Prrx1*-Cre mice to specifically delete the LSD1 gene in limb bud mesenchymal progenitors (*Lsd1*^*Prrx1−/−*^) [30]. Due to the severe limb phenotype and lower body weight [30], *Lsd1*^*Prrx1−/−*^ mice were analysed at 4 weeks of age to minimize the suffering of the animal. Hematoxylin and eosin (H&E)-stained TA muscles revealed substantial decrease of myofiber cross-sectional area (CSA) and altered fibre size distribution in *Lsd1*^*Prrx1−/−*^ mice (Fig. 4D–F). CSA measurement revealed a higher proportion of small diameter fibres in *Lsd1*^*Prrx1−/−*^ mice, while larger fibres were less represented relative to control (Fig. 4F), suggesting that targeted deletion of LSD1 in the limb bud progenitors resulted in defective muscle development and postnatal growth. Collectively, these data support the hypothesis that both, RCOR1 and LSD1, are important for myoblast differentiation.

### RCOR1 regulates myoblast proliferation

**Fig. 5:**
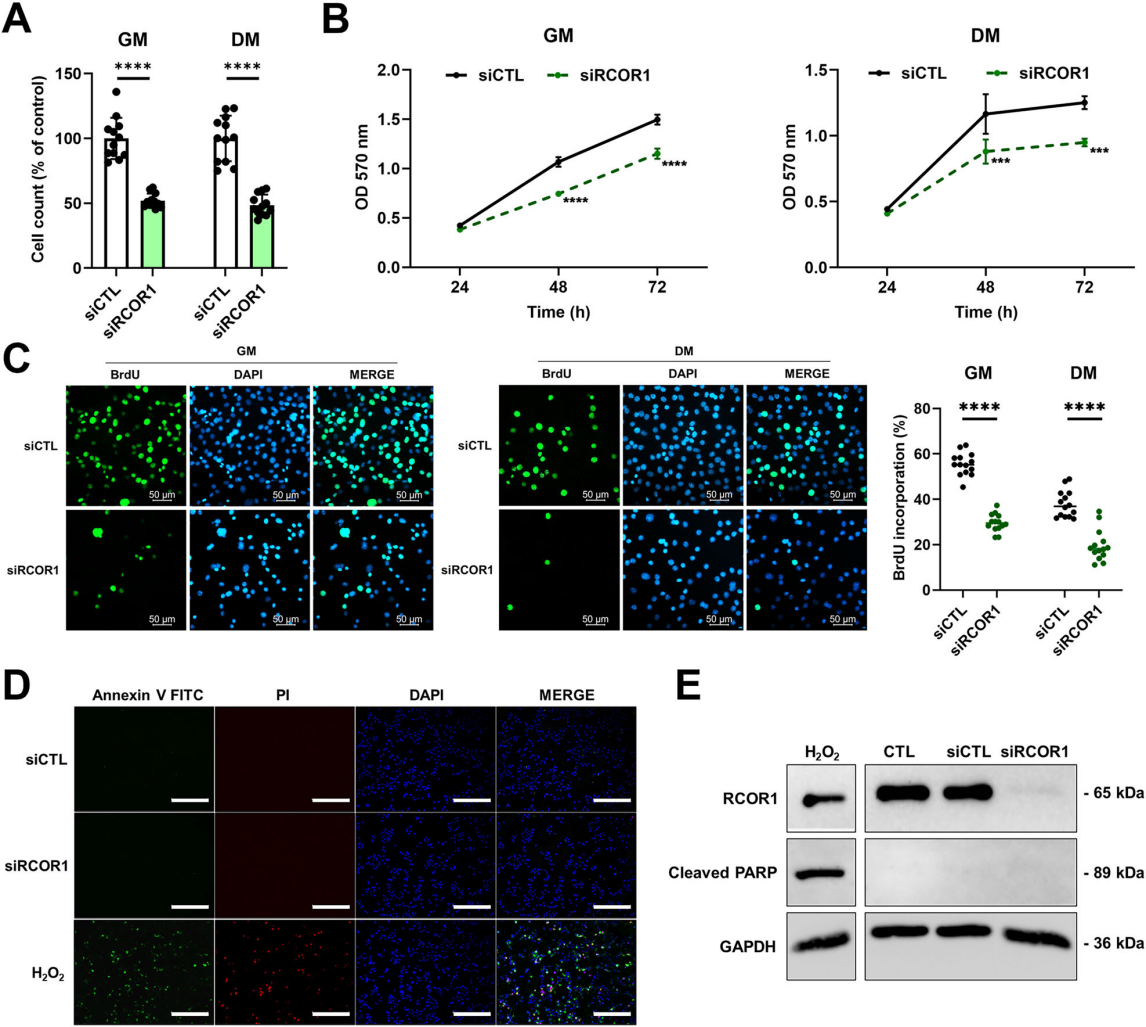
RCOR1 regulates myoblast proliferation. C2C12 cells transfected with control siRNA (siCTL) or RCOR1 siRNA (siRCOR1) were cultured in growth medium (GM) and then switched to fresh GM or differentiation medium (DM) for an additional 1 day. **A** DAPI-stained nuclei were counted in cells cultured in GM and DM. **B** Proliferation analysis was assessed by MTT assay at 24, 48 and 72 h in GM and DM. **C** After 1 day in GM or DM, cells were stimulated with GM or DM containing 10 µM BrdU for 4 h and then immunostained for the detection of BrdU incorporation (green BrdU; blue DAPI). The percentage of the number of BrdU-positive cells was calculated. Scale bars indicate 50 µm. **D** Cell death of transfected C2C12 cells cultured in DM for 1 day was quantified by Annexin V and propidium iodide (PI) staining (scale bar 450 µm) and **E** immunoblotting of cleaved PARP protein levels. For positive control, cells were treated with pro-apoptotic agent H_2_O_2_ (1 mM). Pictures are one representative of 3 independent experiments. Data are presented as means ± SD, ***P < 0.001, ****P < 0.0001; Student’s *t*-test except in **B** a One-way ANOVA was performed.

Differentiation and proliferation of skeletal muscle cells are mutually exclusive and highly regulated processes. Since cell cycle arrest is critical for muscle differentiation, we examined the effect of RCOR1 on C2C12 myoblast proliferation. Silencing RCOR1 reduced the number of cells cultured in both, growth and differentiation medium (Fig. 5A). Consistently, the metabolic rate, as measured by the MTT assay, decreased in siRCOR1-transfected myoblasts relative to controls (Fig. 5B). Next, we performed BrdU incorporation assay in C2C12 myoblasts and primary myoblasts and observed a decreased percentage of BrdU-positive nuclei upon RCOR1 knockdown in both, growth and differentiation medium relative to control cells (Figs. 5C and S5A), indicating a decrease in proliferation in the absence of RCOR1.

To determine whether the decrease in myoblast proliferation was due to increased apoptosis or altered cell cycle regulation, we performed Annexin-V-FITC/PI and DAPI staining. Immunofluorescence analysis revealed no difference between siCTL and siRCOR1-transfected cells and neither group showed apoptotic cell death (Fig. 5D). This result was further confirmed by immunoblotting of cleaved PARP, another marker of apoptosis (Fig. 5E).

**Fig. 6:**
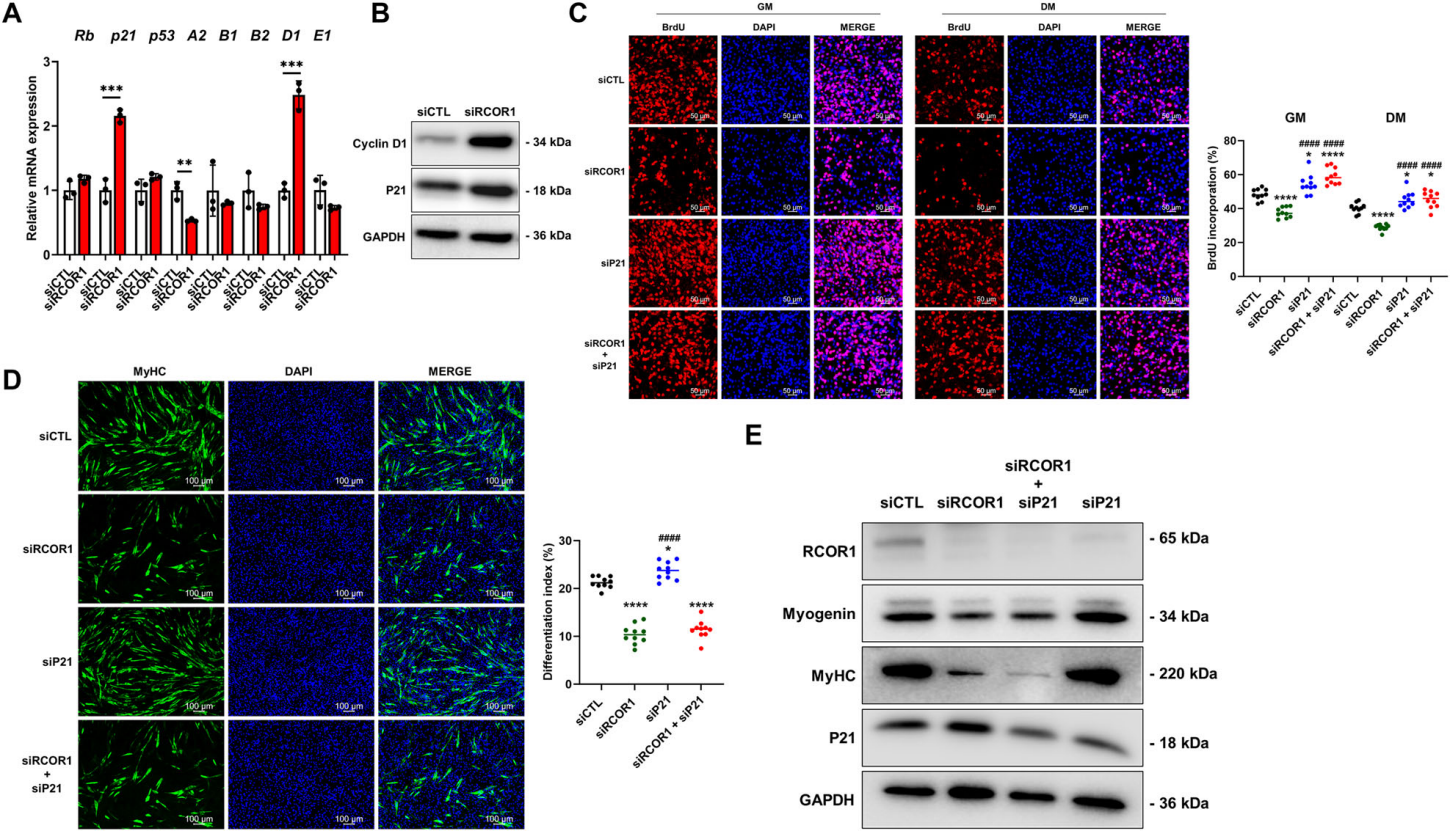
P21 is required for the RCOR1-mediated effect on myoblast proliferation. C2C12 cells transfected with control siRNA (siCTL) or RCOR1 siRNA (siRCOR1) were cultured in a growth medium (GM) and then switched to a fresh differentiation medium (DM) for an additional 1 day. **A** qPCR analysis of cell cycle regulators *Rb*, *p21*, *p53*, *Cyclin A2 (A2)*, *Cyclin B1* (*B1)*, *Cyclin B2 (B2)*, *Cyclin D1 (D1)* and *Cyclin E1 (E1)*. **B** Immunoblotting was performed to detect protein levels of Cyclin D1 and P21 as well as GAPDH as loading control. **C–E** C2C12 cells transfected with a combination of 2 siRNAs including control siRNA (siCTL), RCOR1 siRNA (siRCOR1) or P21 siRNA (siP21) were cultured in GM and then switched into fresh GM or DM for additional 1 day. **C** After 1 day in GM or DM, cells were stimulated with GM or DM containing 10 µM BrdU for 4 h and immunostained for the detection of BrdU incorporation (red BrdU; blue DAPI). The percentage of the number of BrdU-positive cells was calculated. Scale bars indicate 50 µm. **D** Terminally differentiated myotubes transfected with siCTL, siRCOR1, siP21 or siRCOR1 + siP21 were visualized by anti-MyHC immunofluorescent staining (green, MyHC; blue, DAPI) at day 3 in DM. The differentiation index was counted. Scale bars indicate 100 µm. **E** Immunoblotting was performed to detect protein levels of RCOR1, Myogenin, MyHC and P21 at day 3 in DM. GAPDH was used as a loading control. Pictures are one representative of 3 independent experiments. Data are presented as means ± SD. *P < 0.05; **P < 0.01, ***P < 0.001, ****P < 0.0001 relative to corresponding siCTL; ^####^*P* < 0.0001 relative to siRCOR1 in panels C and D; One-way ANOVA except in (**A**) a Student’s *t*-test was performed.

Cell cycle progression is controlled by the activity of cyclins and their associated cyclin-dependent kinases (CDKs) whose activities are negatively regulated by CDK inhibitors (CKIs) [31]. We analysed the expression of several cell cycle regulators (*cyclin A2* (*Ccna2)*, *cyclin B1* (*Ccnb1*), *cyclin B2* (*Ccnb2*), *cyclin D1* (*Ccnd1*), *cyclin E* (*Ccne1*), *RB Transcriptional Corepressor 1* (*Rb1*), *Cyclin dependent kinase inhibitor 1a* (*Cdkn1a* or *p21*) and *p53*) by qPCR in C2C12 myoblasts. siRCOR1-transfected myoblasts exhibited a reduction of cyclin A2, while cyclin D1 increased (Fig. 6A and B). Consistent with reduced proliferation, RCOR1 knockdown increased the mRNA and protein abundance of P21, which is a negative regulator of the cell cycle (Fig. 6A and B). Interestingly, silencing of LSD1 failed to affect C2C12 myoblast proliferation as determined by BrdU incorporation assay and protein expression of P21 (Fig. S5B and C), indicating that LSD1 is not essential for myoblast proliferation. Based on these results, we propose that RCOR1 is required for normal myoblast proliferation by inhibiting the expression of P21. To test this hypothesis, C2C12 cells were treated with siRNAs targeting RCOR1 and P21 alone or in combination, or control siRNAs. The knockdown efficiency was confirmed by immunoblot analysis (Fig S5D). Notably, combined silencing of RCOR1 and P21 increased proliferation relative to siCTL and siRCOR1 -transfected cells, comparable to P21 knockdown alone (Fig. 6C). This suggests that P21 deficiency is able to rescue the RCOR1-mediated proliferation defect in C2C12 myoblasts. We then tested whether combined silencing of RCOR1 and P21 could also rescue myoblast differentiation. However, combined inhibition of RCOR1 and P21 did not affect myoblast differentiation index or differentiation-associated protein expression compared to control or RCOR1 silencing alone (Fig. 6D and E). Taken together, these results support the notion that RCOR1 regulates myoblast proliferation by modulating the levels of P21. However, while silencing P21 rescued the cell proliferation defect of RCOR1-deficiency it failed to restore differentiation, suggesting that these two processes are mediated through independent mechanisms.

### RCOR1 binds with LSD1 and MyoD in myoblasts and regulates LSD1 stability via ubiquitination

**Fig. 7:**
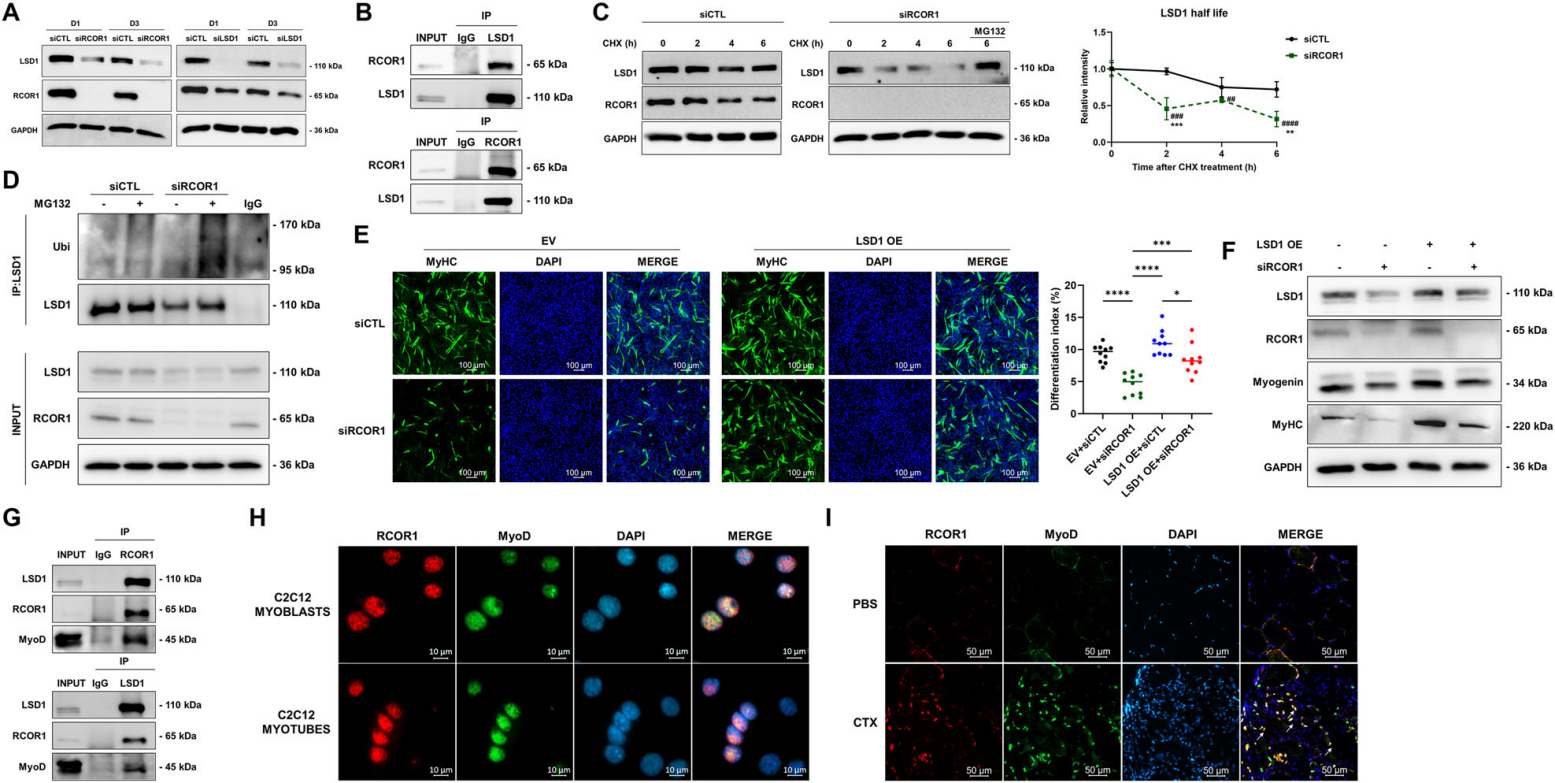
RCOR1 binds with LSD1 and MyoD in myoblasts and regulates LSD1 stability via ubiquitination. **A** C2C12 cells transfected with control siRNA (siCTL), RCOR1 or LSD1 siRNA (siRCOR1; siLSD1) were cultured in growth medium (GM) and induced to differentiate by switching to differentiation medium (DM) for 3 days. Immunoblot was performed to detect protein levels of RCOR1 and LSD1 at day 1 and day 3 in DM. **B** C2C12 cells were cultured in GM and induced to differentiate by switching to DM for 1 day. Cell lysates were immunoprecipitated with anti-LSD1 antibody, anti-RCOR1 antibody or rabbit normal IgG. For immunoblotting anti-LSD1 and anti-RCOR1 antibodies were used. The percentage of input used is 10%. **C** C2C12 cells were transfected with siCTL or siRCOR1 and 48 h later, cells were treated with cycloheximide (CHX) (100 µg/mL) for indicated time intervals. siRCOR1-transfected cells were treated with protease inhibitor MG132 (20 μM) in the presence of CHX as indicated for 6 hours before harvest. Immunoblotting was used to detect and quantify protein stability and change in the level of LSD1. **D** C2C12 cells were transfected with siCTL or siRCOR1 and 48 h later, cells were treated with protease inhibitor MG132 (10 μM) for 6 h before harvest. Cell lysates were immunoprecipitated with anti-LSD1 antibody or rabbit normal IgG. For immunoblotting anti-ubiquitin (Ubi), anti-LSD1, anti-RCOR1 and anti-GAPDH antibodies were used. The percentage of input used is 10%. **E** C2C12 cells were transfected with pCMV3-Flag-mKDM1A plasmid (LSD1 OE) or with empty vector (EV) as a control for 24 h, and subsequently with siCTL or siRCOR1 for an additional 24 h. Cells were then switched into fresh DM for an additional 3 days. Terminally differentiated myotubes were visualized by anti-MyHC immunofluorescent staining (green, MyHC; blue, DAPI) at day 3 in DM. The differentiation index was counted. Scale bars indicate 100 µm. **F** Immunoblotting was performed to detect protein levels of LSD1, RCOR1, Myogenin and MyHC at day 3 in DM. **G** C2C12 cells were cultured in GM and induced to differentiate by switching to DM for 1 day. Cell lysates were immunoprecipitated with anti-LSD1 antibody, anti-RCOR1 antibody or rabbit normal IgG. For immunoblotting was used anti-MyoD antibody. The percentage of input used is 10%. **H** C2C12 cells were cultured in GM and induced to differentiate by switching to DM for 3 days. Immunofluorescence staining for RCOR1 (red) and MyoD (green) was performed in myoblasts and myotubes (blue, DAPI). Scale bar indicates 10 µm. **I** Upon cryosection, immunofluorescence staining for RCOR1 (red), MyoD (green) and DAPI (blue) in TA muscles was performed at day 4 following CTX injury. Scale bar indicates 10 µm. Pictures are one representative of 3 independent experiments or mice. Data are presented as means ± SD, *P < 0.05, **P < 0.01; ***P < 0.001, ****P < 0.0001 relative to corresponding siCTL; ^##^*P* < 0.01, ^###^*P* < 0.001, ^####^*P* < 0.0001 relative to siRCOR1 0 h in (**C**); One-way ANOVA was performed.

RCOR1 is an important binding partner of LSD1 within the CoREST complex and is required for LSD1 catalytic activity and nucleosomal substrate binding [9, 18, 32]. Therefore, we evaluated LSD1 expression following RCOR1 silencing and detected a remarkable decrease of LSD1 protein abundance (Fig. 7A), with no effect on *Lsd1* mRNA expression (Fig. S6A), suggesting post-transcriptional regulation. This was further confirmed by immunofluorescence analysis showing the loss of LSD1 staining in the myoblasts following RCOR1 silencing (Fig. S6B). Upon silencing of LSD1, RCOR1 protein decreased (Fig. 7A), suggesting that RCOR1 and LSD1 interaction is required for the stability of both binding partners.

To explore whether RCOR1 binds with LSD1 in C2C12 cells, we performed co-immunoprecipitation (Co-IP) experiments. As shown in Fig. 7B, we confirmed the interaction between endogenous RCOR1 and LSD1 indicating that the LSD1-RCOR1 complex is present in C2C12 myoblasts during early stages of differentiation. In order to examine whether RCOR1 affects LSD1 stability we transfected C2C12 myoblasts with siRCOR1 and siCTL and after 48 h treated the cells with 100 µM CHX to inhibit protein synthesis. Following CHX treatment, endogenous LSD1 drastically decreased over time in siRCOR1-transfected myoblasts relative to control (Fig. 7C). These data indicate that LSD1 protein degraded faster in the absence of RCOR1. Since previous studies suggested that LSD1 is subjected to proteasome degradation [9, 33], siRCOR1-transfected myoblasts were simultaneously treated with CHX and protease inhibitor MG132. Endogenous LSD1 levels remained unchanged following MG132 treatment, indicating that LSD1 is prone to proteasomal degradation in RCOR1-deficient cells (Fig. 7C). To further investigate whether the loss of RCOR1 promotes the ubiquitination of LSD1, we performed immunoprecipitation of LSD1 from siRCOR1 and siCTL -transfected myoblasts treated with MG132 for 6 h. The data showed a marked increase in ubiquitinated LSD1 in RCOR1-deficient cells following MG132 treatment (Fig. 7D). Together, these results indicate that RCOR1 effect on LSD1 stability is mediated through the ubiquitination of LSD1.

Next, we asked whether the exogenous expression of LSD1 could rescue the compromised myogenic differentiation caused by the loss of RCOR1. To address this question, pCMV3-Flag-mKDM1a plasmid (LSD1 OE) or respective empty vector (EV) were transiently transfected into C2C12 myoblasts. The efficacy of LSD1 overexpression was confirmed by immunoblotting (Fig. S7). The cells were then transfected with control and RCOR1 siRNA, after which the cells were induced to differentiate for up to 3 days. C2C12 cells transfected with the control plasmid and si-CTL differentiated normally (Fig. 7E). In contrast, RCOR1 siRNA transfection suppressed differentiation in C2C12 cells (Fig. 7E). Interestingly, transient LSD1 overexpression in siRCOR1-transfected C2C12 cells abolished the inhibitory effect of RCOR1 depletion on myogenic differentiation, as shown by increased differentiation index (Fig. 7E). Furthermore, protein expression of myogenin and MyHC was restored to levels of control myoblasts (Fig. 7F). These data support the notion that degradation of LSD1 is most likely responsible for the RCOR1-mediated effect on myogenic differentiation.

Upon the initiation of differentiation, MyoD activates the expression of muscle-specific genes and the formation of differentiated myotubes. Furthermore, MyoD has been shown to interact with many transcription factors to control muscle gene expression and muscle differentiation [34]. Since RCOR1 inhibition suppressed the expression of known MyoD target genes such as myogenin, we investigated a possible interaction between RCOR1 and MyoD in myoblasts. LSD1 was previously shown to interact with MyoD [12], allowing us to hypothesize that RCOR1 also associates with MyoD and consequently contributes to the regulation of MyoD-dependent transcription. We performed co-IP experiments during the onset of differentiation in C2C12 cells and confirmed the association between endogenous RCOR1 and MyoD (Fig. 7G). Furthermore, RCOR1 and MyoD were colocalized in the nuclei of C2C12 myoblasts and myotubes (Fig. 7H). MyoD is expressed in activated and proliferating SCs upon muscle injury [35]. To assess whether RCOR1 and MyoD are co-expressed in injured muscle, we performed immunofluorescence on regenerating muscle sections. Indeed, the majority of MyoD-positive mononucleated cells were also RCOR1-positive, indicating that SCs express RCOR1 as they become activated upon injury (Fig. 7I). Together, our results indicate that RCOR1 is critical for the differentiation capacity of myoblasts and acts through stabilizing LSD1. Moreover, RCOR1 can associate with LSD1 and MyoD in myoblasts and SCs and potentially mediate MyoD-dependent transcription activation of myogenin.

### Loss of RCOR1 impairs muscle regeneration in vivo

**Fig. 8:**
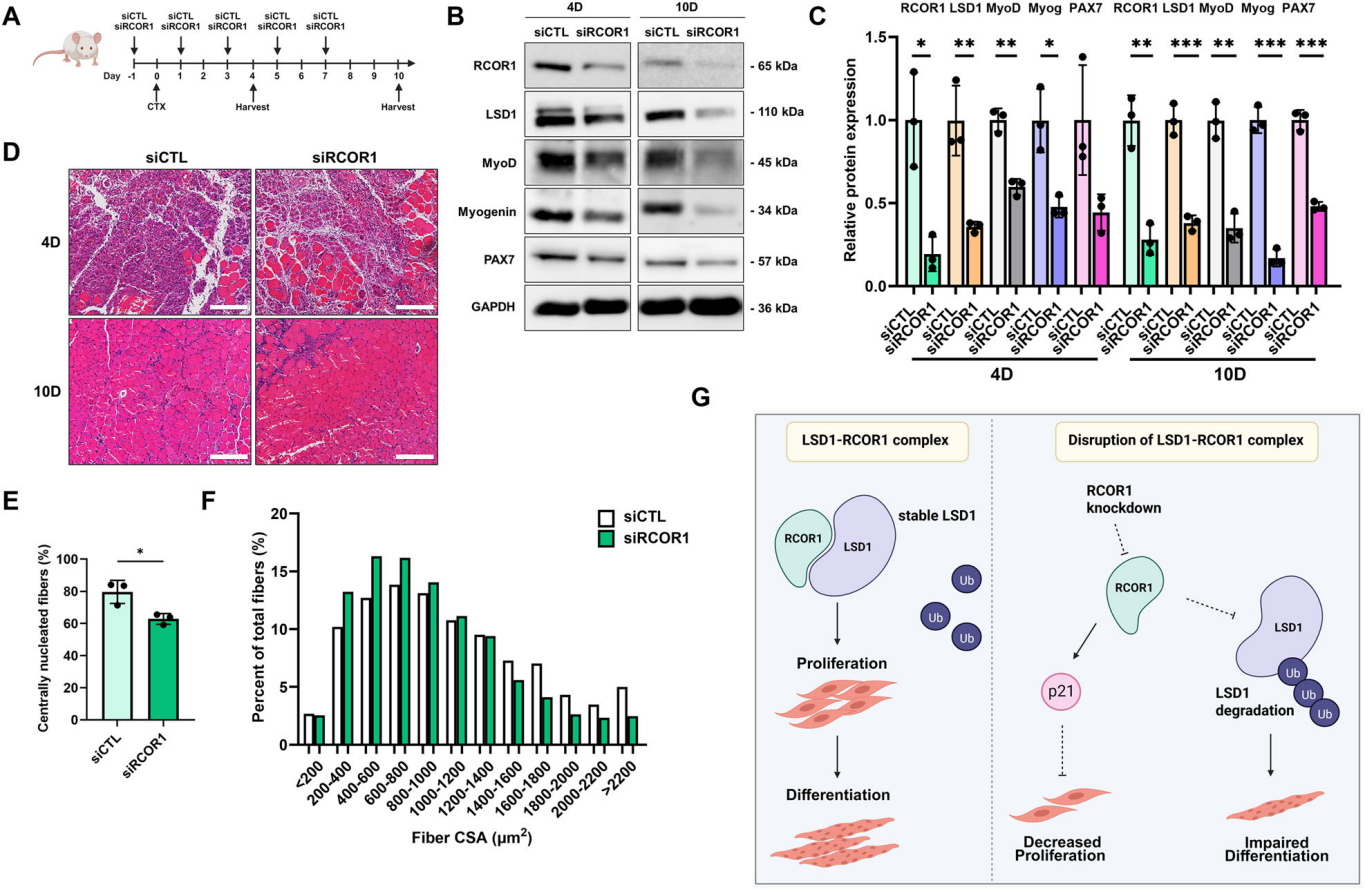
Loss of RCOR1 impairs muscle regeneration. **A** Schematic diagram of siRCOR1 mediated silencing of RCOR1 in the CTX injury muscle model. Mouse tibialis anterior (TA) muscles were treated with siRCOR1 or siCTL followed by CTX injection to induce muscle injury. At days 4 and 10 following injury, muscles were harvested for analysis. *n* = 3 mice per group. **B** Immunoblot and **C** quantification of protein abundance of RCOR1, LSD1, MyoD, Myogenin and PAX7 at days 4 and 10 following injury. **D** TA muscles were stained by hematoxylin and eosin (H&E) and analysed for **E** percent of centralized myonuclei and **F** percent distributions based on CSA (μm^2^) at day 10. Scale bars indicate 200 µm. **G** Proposed simplified model for RCOR1 function in myoblast proliferation and differentiation. Data are presented as mean ± SD; *P < 0.05, **P < 0.01; ***P < 0.001 relative to siCTL; Student’s *t*-test was performed.

To further examine the role of RCOR1 in muscle regeneration in vivo, we depleted RCOR1 in the TA muscle during CTX-mediated muscle regeneration. To ensure that RCOR1 was inhibited throughout the experiment, siRCOR1 and siCTL were injected every 2 days into TA muscle during regeneration, and muscles were harvested at days 4 and 10 for analysis (Fig. 8A). Effective knockdown of RCOR1 protein was confirmed by immunoblot and was accompanied with reduction of LSD1 (Fig. 8B and C), consistent with observations in vitro (Figs. 3C and S3C). Both, siRCOR1 and siCTL-treated TA muscles displayed newly formed muscle fibres characterized by centralized nuclei (Fig. 8D). However, the number of myofibers containing centrally located nuclei was reduced in siRCOR1 muscles at 10 days following CTX injection (Fig. 8E). We also measured CSA of regenerative myofibers and observed a shift toward smaller sized fibres in TA muscles treated with siRCOR1 (Fig. 8F). Muscle regeneration involves activation and proliferation of SCs and their further differentiation to myocytes before maturing to myofibers. To investigate whether impaired muscle regeneration is associated with a reduced pool of satellite cells, we analysed the expression of SC marker Pax7 and found that RCOR1 deficiency in TA muscles reduced the expression of PAX7 (Fig. 8B and C). Furthermore, the differentiation capacity of SCs was impaired, as shown by suppressed expression of myogenic markers MyoD and myogenin in siRCOR1-depleted muscles relative to control (Fig. 8B and C). These data strongly indicate that RCOR1 contributes to muscle regeneration following injury.

## Discussion

Our study delineates a novel role of RCOR1 as a regulator of myogenesis and muscle regeneration. Loss of RCOR1 remarkably impaired myoblast differentiation in vitro and muscle regeneration in vivo, caused by the reduced number and decreased myogenic aptitude of SCs. Mechanistically, RCOR1 was found to interact with LSD1 and myogenic regulatory factor MyoD and contribute to LSD1 protein stability in myoblasts. Moreover, RCOR1 was required for both the proliferative and differentiation potential of myoblasts, by regulating P21 expression and LSD1 proteasomal degradation, respectively (Fig. 8G). Collectively, our data provide first insights into the role of RCOR1 in skeletal muscle differentiation and regeneration.

RCORs are best known as core components of the chromatin-modifying CoREST repressor complex, which also includes LSD1 and HDAC1/2 among several other subunits [23, 27, 36]. Within this complex, RCOR proteins facilitate LSD1-mediated demethylation of nucleosomes and therefore are essential for LSD1 activity [9, 17, 32]. In recent years, LSD1 was identified as a key epigenetic regulator of myoblast differentiation and skeletal muscle regeneration [12, 15, 16]. This prompted us to investigate whether members of the RCOR family could also have a role in these myogenic mechanisms. Although RCORs have been shown to play a role in the development and homoeostasis of different tissues, nothing is known about their function in muscle cells. To address this, we first determined that C2C12 cells and primary mouse myoblasts express all three RCOR family members. *Rcor1* was expressed at a higher level than *Rcor2* and *Rcor3*, which could explain its dominant role in myoblasts. Protein levels of RCOR1 and LSD1 are found to be interdependent [9, 37]. Here, we showed that RCOR1 forms a complex with LSD1 in myoblasts. Moreover, RCOR1 knockdown resulted in a decrease of LSD1 protein in myoblasts, while *Lsd1* mRNA expression was unaffected, suggesting post-transcriptional regulation. Interestingly, silencing RCOR1 greatly increased LSD1 ubiquitination, supporting our hypothesis that RCOR1 is critical for controlling myogenic differentiation via LSD1 stabilization in myoblasts. This was confirmed by rescue experiments, showing that LSD1 overexpression reversed the impaired differentiation of RCOR1-deficient myoblasts. Our data confirm that the effect of RCOR1 on myogenic differentiation is specifically dependent on LSD1. Interestingly, LSD1 dysregulation is observed in human patients with neurodegenerative diseases and neuromuscular disorders [38, 39]. Thus, targeting the LSD1 protein, not only its demethylase activity, could provide some alternative strategies to better target these diseases.

Previous studies reported that LSD1 levels increase during C2C12 myoblast differentiation [12, 15]. Contrary to these reports, we found that both LSD1 and RCOR1 protein levels were highest in proliferating myoblasts and decreased during differentiation to myotubes. Tosic et al. showed that LSD1 ablation not only delays skeletal muscle regeneration but also switches the fate of SCs towards brown adipocytes [16]. Thus, it is tempting to suggest that elevated RCOR1 and LSD1 levels in undifferentiated myoblasts are required to restrict the adipogenic potential and direct precursor cells toward the myoblast phenotype. RCOR1 is important for balancing the proliferation and differentiation during brain development, as deletion of RCOR1/2 generated a large number of neural progenitors at the expense of differentiated neurons [40]. In addition, a decrease in RCOR1 and LSD1 was observed during neuronal differentiation, suggesting that downregulation was necessary for the differentiation process [41]. Given their nuclear localization and similar expression pattern during myoblast differentiation, our data suggest that RCOR1 and LSD1 could act together with MyoD at early stages of differentiation. LSD1 either physically interacts with MyoD and binds to the myogenin promoter which leads to the activation of myogenic genes or modulates MyoD expression which regulates the entry of myoblasts into the differentiation process [12, 15]. Accordingly, we show that RCOR1 and LSD1 depletion in myoblasts severely impairs myogenic differentiation and downregulates the expression of important MyoD target genes such as myogenin, while MyoD remains unaltered. Therefore, we speculated that RCOR1 and LSD1 work together in myogenesis, at least partially through regulating MyoD-dependent transcription activity. Indeed, we confirmed that MyoD binds both RCOR1 and LSD1 in differentiated C2C12 myoblasts, suggesting that they are recruited to the myogenin promoter by MyoD to facilitate muscle-specific gene expression.

Our work suggests that RCOR1 is required to maintain the proliferation of myoblasts. Although myoblast proliferation and differentiation are mutually exclusive [42], we found both processes to be impaired by RCOR1 depletion, suggesting that they could be regulated through different mechanisms. Consistent with the cell cycle arrest phenotype, RCOR1-deficiency caused an increase of CDK inhibitor P21. Combined silencing of RCOR1 and P21 rescued the RCOR1-mediated proliferation defect, but failed to rescue myogenic differentiation, indicating that impaired proliferation observed in the absence of RCOR1 is a P21-dependent process. These results suggest that RCOR1 effects on proliferation and differentiation occur by distinct mechanisms and that only the former is P21-dependent. Although previous studies showed that P21 is transcriptionally upregulated by LSD1 [43], LSD1 knockdown had no effect on the proliferation rate or the expression of P21 in myoblasts. This discrepancy in proliferation suggests that the role of RCOR1 in cell proliferation might be LSD1-independent and regulated by other subunits of the CoREST complex. Although RCORs have not yet been identified in complexes without LSD1 in mammals, in *Drosophila* the RCOR homologue was present in LSD1-free complexes [44]. Furthermore, LSD1 has also been found in the NuRD [45] complex, which does not contain RCORs. This complex possesses both histone deacetylase and demethylase activity and the coexistence of LSD1/NuRD and LSD1/CoREST complexes has been confirmed. Equally important was the observation that siRNA-mediated inhibition of RCOR1 and LSD1 resulted in high expression of Cyclin D1, a cell cycle protein whose downregulation is required for the initiation of differentiation [46, 47]. Furthermore, inhibition of MyoD function mediated by Cyclin D1 was shown to be unrelated to its role in cell cycle progression [48].

Notably, RCOR1 depletion in muscle also impaired injury-induced muscle regeneration, as evidenced by decreased size of regenerated myofibers. Muscle regeneration is a highly orchestrated process mediated by the activation, proliferation and differentiation of SCs [49]. Therefore, any dysregulation of these different myogenic stages could lead to impaired regeneration. Here, we found RCOR1 to be highly expressed in MyoD-activated SCs during early regeneration upon muscle injury, indicating that RCOR1 promotes SC function. Accordingly, RCOR1-depleted muscles exhibited reduced expression of MyoD and myogenin indicating inhibited myogenic commitment of SCs. This was accompanied by reduced expression of PAX7, which might result from reduced proliferation capacity of SCs in response to RCOR1 depletion, as similar impairment in proliferation was observed in in vitro myoblast culture. In addition, we found decreased LSD1 levels following RCOR1 depletion upon muscle injury in vivo. Therefore, we propose that both RCOR1 and LSD1 participate in SCs myogenic lineage progression. Interestingly, mice with selective LSD1 ablation in Pax7-positive SCs had no differences in morphology and fibre size distribution but experienced decreased regenerative capacity upon muscle injury [16]. However, our results demonstrate that LSD1 has an important role in muscle development and postnatal muscle growth, as conditional deletion of LSD1 in limb bud cells resulted in decreased body weight and marked reduction in myofiber size. Thus, it is likely that the difference in the genetic engineering methods accounts for the phenotypic divergence, and that LSD1 has a maturation stage-dependent function in the muscle cell lineage. Previous studies showed that disruption of the *Rcor1* gene caused embryonic lethality [22, 40]. However, a study with zebrafish RCOR1 mutants showed that these animals managed to survive to adulthood [50]. Although surviving adults developed normally and were fertile, RCOR1 mutants showed locomotive impairment and were hypoactive relative to control animals. Muscle development and regeneration were not evaluated, but these data suggest that inhibition of RCOR1 could lead to defects in skeletal muscle in zebrafish. Taken together, these data suggest that RCOR1 is required for activation and differentiation of SCs during muscle regeneration.

These data are the first evidence for a unique role of RCOR1 in skeletal muscle cells and muscle regeneration. RCOR1 was shown to function in myoblast proliferation and differentiation into myotubes by regulating the expression of cell cycle and muscle-specific genes. Moreover, RCOR1 depletion decreased injury-induced SC activation and differentiation resulting in impaired muscle regeneration. Our findings provide new insights into potentially exploitable therapeutic factors in musculoskeletal health.

## Materials and methods

### Animal studies

Mouse studies were approved by the Finnish Ethical Committee for Experimental Animals (license number #14044/2020), complying with the international guidelines on the care and use of laboratory animals. All mice were age-matched and randomized. Male mice on a C57BL/6JRj background were housed under standard laboratory conditions and were fed ad libitum. The mice were euthanized with CO_2_-asphyxiation followed by sample collection. For the analysis of RCOR1 expression during postnatal muscle development, hindlimb muscles were obtained from mice at postnatal day 1 (P1), 2 weeks (W2) and 4–6 weeks (W4–6) of age. Neonate mouse pups were euthanized by decapitation. Mice embryos carrying the floxed *Kdm1a/Lsd1* alleles (*Lsd1*^fl/fl^, stock #023969) were obtained from the Jackson laboratory. *Lsd1*^fl/fl^ mice were crossed with *Prrx1-Cre* to create limb bud mesenchyme-specific conditional *Lsd1*-knockout mice (*Lsd1*^Prrx1−/−^), as described previously [30]. Mice were analysed at 4 weeks of age.

### Cardiotoxin injury

Cardiotoxin (CTX; Latoxan, France) was dissolved in sterile saline to a final concentration of 100 µM. Male 8–10 weeks old C57BL/6JRj mice were anaesthetised with isoflurane inhalation and provided with analgesia (0.1 mg/kg buprenorphine) to alleviate pain. Right tibialis anterior (TA) muscles were intramuscularly injected with 20 μl of 20 µM CTX solution with a hypodermic needle, while left TA muscles were injected with sterile saline only. Regenerating TA muscles were isolated 4, 7, and 10 days following CTX injection.

### Intramuscular transfection of siRNAs

Intramuscular transfection of siRNAs (5 µg) was performed using an in vivo-jetPEI kit (Polyplus) following the manufacturer’s recommendations. PEI/siRNA complexes were formed at an N/P ratio of 8 in equal amounts of 5% glucose solution. After 15 min incubation at RT, the PEI/siRNA complex mixture (20 µl) was injected intramuscularly into mouse TA muscles with a hypodermic needle. Hindlimbs of 8–10-week-old male mice were shaved and cleaned with 75% alcohol. The mixture containing si-RCOR1 was injected into the right TA muscle, while the mixture containing si-CTL was injected into the left TA muscle as a negative control. Regenerating TA muscles were isolated at 4 and 10 days following CTX injection.

### TA muscle histology

For histological analyses, freshly isolated TA muscles were fixed in formalin, dehydrated by graded ethanol, embedded in paraffin, and cut into 5 μm sections. The sections were deparaffinized, rehydrated, and stained with haematoxylin & eosin. Cross-sectional areas (CSA) and diameters of TA fibres were measured using ImageJ software. Minimum of 1000 muscle fibres was measured for each sample. For immunofluorescence studies, regenerating TA muscles were flash-frozen in liquid nitrogen-cooled isopentane. Frozen blocks were embedded in Optimal Cutting Temperature (O.C.T.) compound and sectioned at 8 μm using a cryostat microtome. Following fixation with 4% formaldehyde for 15 min, antigen retrieval was performed by incubating muscle sections in boiling 10 mM citrate buffer pH 6 for 10 min. Then, samples were permeabilized with 0.5% Triton X-100 for 20 min, blocked with 3% BSA for 1 h and incubated with AffiniPure Fab Fragment Goat Anti-Mouse IgG (H + L) (Jackson ImmunoResearch) in PBS (20 µg/mL) for 1 h. Primary antibodies were incubated overnight at 4 °C, followed by incubation with Fluor Alexa 488- and 594-conjugated secondary antibodies (Jackson ImmunoResearch Laboratories). DAPI was used to visualize nuclei. PBS washes were performed between each step. Unless indicated otherwise, all incubations were carried out at RT.

### Cell culture

All cells were cultured in a 37 °C incubator with 5% CO_2_. C2C12 myoblasts (ATCC) were cultured in high-glucose Dulbecco’s modified Eagle medium (DMEM; Sigma-Aldrich), supplemented with 10% foetal bovine serum (FBS; Gibco) and 1% penicillin–streptomycin (Gibco) (Growth Medium, GM). Once cells reached 80–90% confluence, differentiation was induced by DMEM supplemented with 2% horse serum (HS, Gibco) and 1% penicillin/streptomycin (Differentiation Medium, DM). The medium was changed every other day.

Primary myoblasts were isolated from the hindlimbs of 7–9-week-old C57BL/6JRj male mice as described previously [51]. Briefly, muscles were minced using scissors and digested with 400 U/ml of collagenase II (Gibco) solution for 1 h at 37 °C. To remove tissue debris and large infiltrating cells, the digested tissue suspension was passed through a 70 µm cell strainer and an additional 30 µm filter. Cells were plated on 10 cm Matrigel-coated dishes in Ham’s F10 nutrient mixture (Sigma Aldrich) with 20% FBS, 10 ng/mL fibroblast growth factor 2 (FGF2; PeproTech) and 1% penicillin–streptomycin. To remove fibroblasts, cells were incubated with a small amount of PBS at room temperature (RT) for 5 min. The detached cells were collected with Ham’s F10 nutrient mixture and plated on a new Matrigel-coated dish. This was repeated until no fibroblasts were observed in the culture. Differentiation was induced with DMEM supplemented with 5% HS and 1% penicillin-streptomycin when cells reached 90% confluency. The medium was changed every other day.

### siRNA-mediated gene knockdown

C2C12 cells and primary mouse myoblasts were transfected with SMARTpool siRNA oligonucleotides (Dharmacon) using Lipofectamine RNAImax (Invitrogen), according to the manufacturer’s instructions. Briefly, RNAImax–siRNA complexes were added into each well to yield a final concentration of 20 nM siRNAs. Differentiation was induced 24 h after and cells were harvested at indicated time points. The siRNAs used in this study were SMARTpools of ON-TARGETplus Non-targeting siRNA (siCTL), ON-TARGETplus Mouse LSD1 siRNA (siLSD1), ON-TARGETplus Mouse RCOR1 siRNA (siRCOR1) and ON-TARGETplus Mouse cyclin-dependent kinase inhibitor 1A siRNA (siP21). For the double knockdown, a mixture of 2 siRNAs (siCTL, siRCOR1, sip21; 20 nM per siRNA) was employed.

### Plasmids and transfection

For LSD1 overexpression experiments, C2C12 cells were transfected with 1 µg of pCMV3-Flag-mKDM1A plasmid (Sino Biological) or 1 µg of corresponding control plasmid (empty vector) as a negative control using Lipofectamine 3000 (Invitrogen), according to the manufacturer’s instructions.

### Cell proliferation assay

Cell proliferation was measured by CellTiter 96® Non-Radioactive Cell Proliferation Assay Kit (MTT; Promega) according to the manufacturer’s instructions. Briefly, C2C12 myoblasts were plated at 2000 cells per well in 96-well plates and assayed at 24, 48- and 72-h post plating. At indicated times, 15 µl of dye solution was added directly to the wells and plates were incubated for 3 h in a humidified 37 °C incubator with 5% CO_2_. Absorbance was measured at 570 nm by using an absorbance microplate reader (Ensight).

### 5‑Bromo‑2‑deoxyuridine (BrdU) assay

5‑Bromo‑2‑deoxyuridine (BrdU) assay was performed with BrdU (Santa Cruz Biotechnology) and anti-BrdU antibody (Santa Cruz Biotechnology). Cells were cultured in a growth medium for 24 h and then switched into a fresh growth medium or differentiation medium. After 24 h cells were stimulated with growth or differentiation medium containing 10 µM BrdU for 4 h prior to harvesting. Cells were fixed, permeabilized, treated with 1 M HCl for 1 h, and incubated overnight at 4 °C with anti-BrdU antibody following incubation with Alexa Fluor-488 conjugated goat anti-mouse IgG (Jackson ImmunoResearch Laboratories). Slides were mounted in the mounting medium for fluorescence with DAPI (Vector Laboratories). The proliferation rate was calculated as the ratio between cells stained with BrdU and those stained with DAPI.

### Apoptosis assay

C2C12 myoblasts were stained with Annexin V FITC/PI kit (Santa Cruz Biotechnology) according to the manufacturer’s instructions. Briefly, cells were incubated with 500 μL 1x binding buffer, 2.5 μg FITC-Annexin V and 50 µl propidum iodide (PI) at RT for 15 min in the dark. The stained samples were detected by fluorescence microscopy.

### RNA isolation and RT-qPCR

Total RNA was isolated using the NucleoSpin RNA Plus kit (Macherey-Nagel) following the manufacturer’s instructions. cDNA synthesis was performed with Sensifast cDNA synthesis kit (Meridian Bioscience) and Quantitative PCR (qPCR) on CFX384 Real-Time PCR Detection System using DyNAmo Flash SYBR Green qPCR Kit (ThermoFisher Scientific). Relative quantification of gene expression was performed by the 2^–ΔΔCt^ method using *Gapdh* as the housekeeping gene for normalization. Primer sequences are shown in Table S1.

### Immunoblotting

Cells were lysed in radioimmunoprecipitation assay (RIPA) buffer (50 mM Tris–HCl, pH 7.4; 0.5% NP-40; 0.25% sodium deoxycholate; 150 mM sodium chloride; 1 mM sodium orthovanadate; 0.5 mM phenylmethylsulfonyl fluoride), while muscle tissues were lysed in Lysis buffer (20 mM Tris–HCl, pH 7.8; 137 mM NaCl; 2,7 mM KCl; 1 mM MgCl_2_; 1% Triton x-100; 10% glycerol; 1 mM EDTA, 1 mM DTT), both supplemented with protease inhibitor cocktail (Roche). Samples were incubated on ice for 30 min and cell debris was separated by centrifugation at 10,000×*g* for 15 min at 4 °C. The supernatant was collected and protein concentration was determined by Bradford colorimetric assay (Bio-Rad). An equal amount of protein was loaded on 12% SDS–PAGE gels and transferred to nitrocellulose membranes. Blocking was performed in Tris‑buffered saline with 5% non‑fat dry milk for 1 h at RT. Membranes were probed with primary antibodies at 4 °C overnight, followed by incubation with horseradish peroxidase (HRP)-linked secondary antibodies (Cell Signaling) for 1 h at RT. The blots were visualized with WesternBright Quantum kit (Advansta) and images were captured with the LAS-4000 Luminescent imager (Fujifilm Life Sciences). Quantification of the density of each band was performed by Image J software. Antibody information is shown in Table S2.

### Immunofluorescence

Cells were rinsed in PBS, fixed in 4% formaldehyde (PFA, Invitrogen) for 15 min and permeabilized in 1% TritonX-100/PBS solution for 20 min at RT. Cells were then blocked for 30 min in PBS/3%BSA/0.05%Triton X-100 blocking solution and incubated with primary antibodies prepared in blocking solution for 2 h RT or overnight at 4 °C. This was followed by 3 washes in PBS and incubation with Alexa Fluor-594-conjugated goat anti-rabbit IgG and Alexa Fluor-488 conjugated goat anti-mouse IgG (Jackson ImmunoResearch Laboratories) for 1 h in the dark. After washing in PBS, slides were mounted in the mounting medium for fluorescence with DAPI (Vector Laboratories). Images were obtained by Axio Imager Z2 (Zeiss) microscope using ZEN software.

### Co-immunoprecipitation

Cells were washed in PBS, lysed in IP buffer (10 mM Tris pH 8, 0.4% NP40, 300 mM NaCl, 10% glycerol, 1 mM DTT, protease inhibitor cocktail (Roche)), passed 10 times through a 25 G needle and incubated for 30 min at 4 °C. Cell lysates were centrifuged at 15,000×*g* for 15 min at 4 °C, supernatants were diluted with one volume of dilution buffer (10 mM Tris pH 8, 0.4% NP40, 5 mM CaCl2) and 10% of chromatin was stored as input. Samples were precleared with Dynabeads Protein G (Thermo Fisher Scientific) for 2 h at 4 °C and then incubated with 3 µg of primary antibody on a rotary platform overnight at 4 °C. The next day, beads were blocked in 5 mg BSA/PBS for 2 h at 4 °C and added to the samples for an additional 2 h at 4 °C. The unbound supernatant was aspirated and the beads were washed with ice-cold diluted IP buffer. Proteins were eluted with SDS sample buffer by heating at 99 °C for 5 min and analysed by immunoblotting.

### Protein stability assay

C2C12 cells were transfected with 20 nM of siCTL or siRCOR1 and after 48 h treated with 100 μg/ml cycloheximide (CHX; Sigma Aldrich) to inhibit protein synthesis. CHX-treated cells were harvested at different time points (0, 2, 4 and 6 h) and processed for immunoblotting with anti-LSD1 (Abcam) and anti-RCOR1 (Abcam) antibodies. Anti-GAPDH antibody (Abcam) was used as an internal control. C2C12 cells were simultaneously treated with proteasomal inhibitor MG132 (20 μM: Santa Cruz Biotechnology) for 6 h and processed for immunoblotting.

### Ubiquitination assay

C2C12 cells were transfected with 20 nM concentration of siCTL and siRCOR1. After 48 h cells were treated for 6 h with 10 µM MG132 and lysed. The samples were immunoprecipitated using Dynabeads Protein G, as previously described. Subsequent immunoblotting was performed using Ubiquitin antibody (Cell Signalling).

### Quantification and Statistics

The differentiation index was calculated as the percentage of nuclei in MyHC-positive cells compared to the total number of nuclei. The fusion index was calculated as the percentage of nuclei in MyHC-positive myotubes with ≥2 nuclei out of the total nuclei. All data were expressed as mean ± standard deviation (SD). No statistical method was used to predetermine sample size, but similar sample sizes were chosen in accordance with previous publications. The exact number of animals used for each figure is indicated in the corresponding figure legend. We did not systematically assess the variance within groups. Statistical differences between groups were determined by unpaired Student’s t-test when comparing two groups or one-way ANOVA with Tukey’s post-hoc test when more than two groups were compared by using GraphPad Prism 10.2.0. All experiments were performed independently at least three times. No samples or animals were excluded from the analysis. Data collection and analysis were not performed blind to the conditions of the experiments. *P* values smaller than 0.05 were considered to be significant (*P < 0.05; **P < 0.01; ***P < 0.001; ****P < 0.0001).

## Supplementary information

Supplementary table 1

Supplementary table 2

Supplemental material

Original immunoblots

## Data Availability

The manuscript does not contain RNA-Seq or other large data sets. Detailed information on antibodies and primers used in the manuscript is provided in the supplemental tables.
