# Supplementary material for: RCOR1 promotes myoblast differentiation and muscle regeneration
Source: Cell Death Discov. 2025 Jul 1;11:298. doi: 10.1038/s41420-025-02568-9 (PMC12217761; doi:10.1038/s41420-025-02568-9)
Supplement: Supplementary file 3 — Supplemental material [file 41420_2025_2568_MOESM3_ESM.pdf]

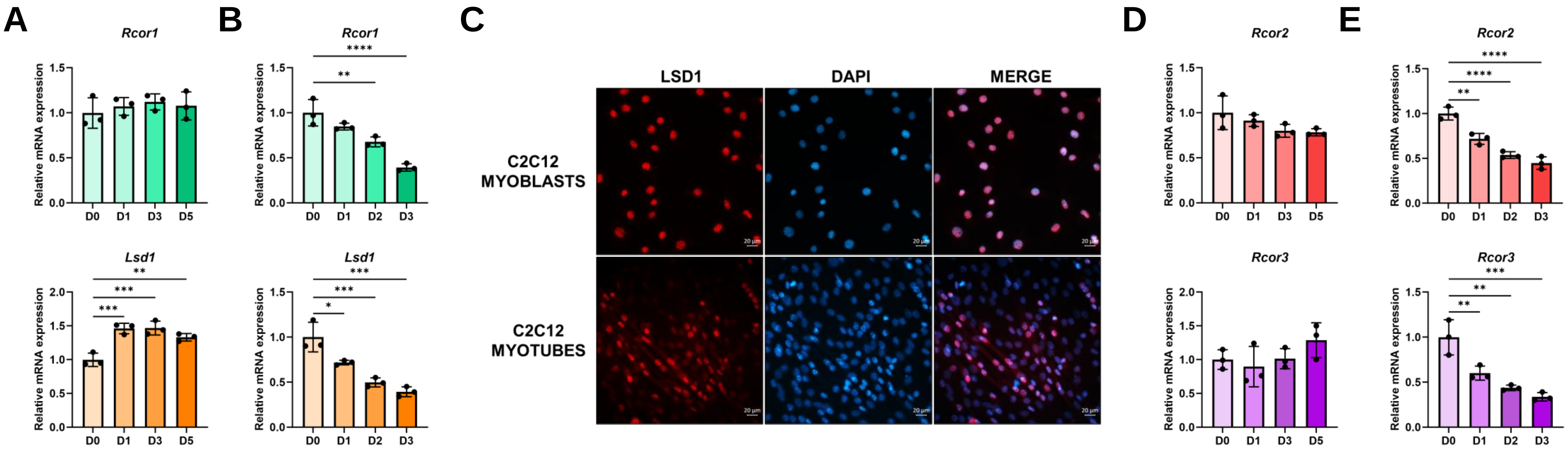

Figure S1. Myoblasts cultured in growth medium (GM) were induced to differentiate by switching to differentiation medium (DM) for 5 days. The expression levels of *Rcor*1 and *Lsd1* were detected by qPCR in (**A**) C2C12 and (**B**) primary mouse myoblasts. (**C**) Immunofluorescence staining for LSD1 in C2C12 myoblasts and myotubes (red, LSD1; blue, DAPI). Scale bar 20 µm. mRNA expression of *Rcor* family members *Rcor2* and *Rcor3* was analysed by qPCR in (**D**) C2C12 and (**E**) primary myoblasts. Pictures are representatives of 3 independent experiments. Data are presented as mean ± SD. *P < 0.05; **P< 0.01; ***P< 0.001; ****P< 0.0001. One-way ANOVA was performed.

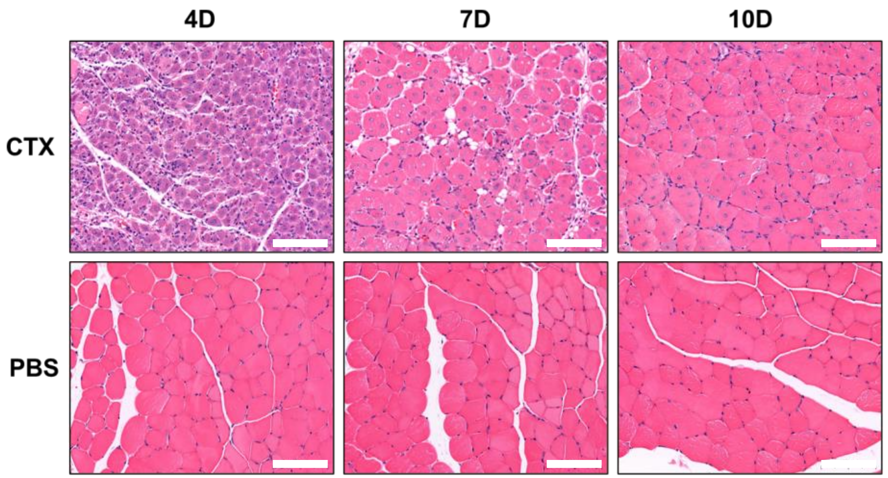

Figure S2. Hematoxylin and eosin (H&E) staining of TA muscle sections collected at 4, 7 and 10 day following CTX or PBS injection. Scale bar 100 µm.

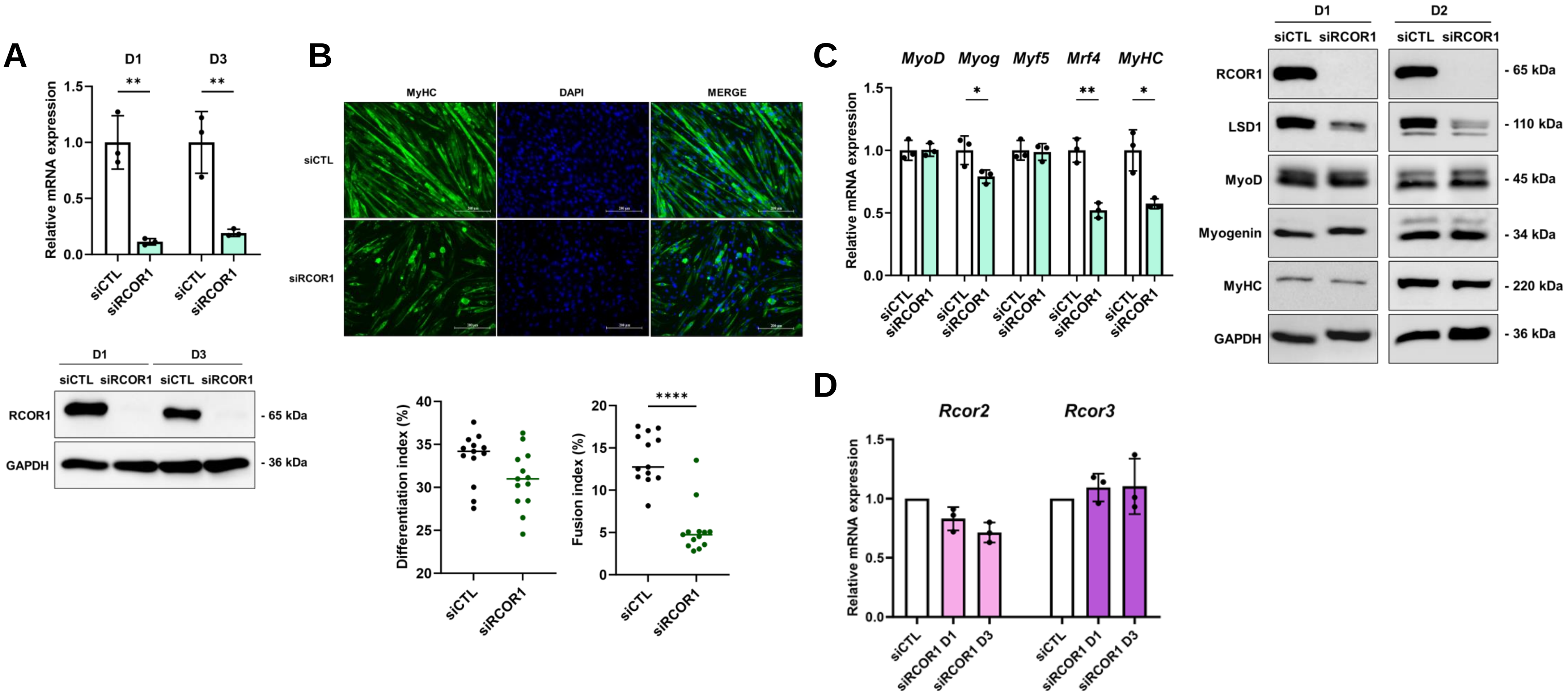

Figure S3. C2C12 cells were transfected with siRCOR1 and siCTL and induced to differentiate for 3 days. (**A**) Knockdown efficiency was determined by qPCR and immunoblotting during 3-days of differentiation. (**B**) Primary myoblasts isolated from mouse limb muscles were transfected with siCTL or siRCOR1 and induced to differentiate for 2 days. Terminally differentiated myotubes were visualized by anti-MyHC immunofluorescent staining (green, MyHC; blue, DAPI) at day 2 in DM. The differentiation index and fusion index were counted. Scale bar 200 µm. (**C**) qPCR and immunoblotting were performed to detect the mRNA and protein levels of RCOR1, LSD1, MyoD, Myogenin, Myf5, Mfr4 at day 1 and MyHC at day 2 in DM. (**D**) mRNA expression of *Rcor2* and *Rcor3* following RCOR1 knockdown in C2C12 myoblasts was analysed by qPCR at day 1 and 3 in DM. Pictures are representatives of 3 independent experiments. Data are presented as means ± SD. *$P < 0.05$; **$P< 0.01$; ****$P< 0.0001$. Student's t-test except in (**D**) a One-way ANOVA was performed.

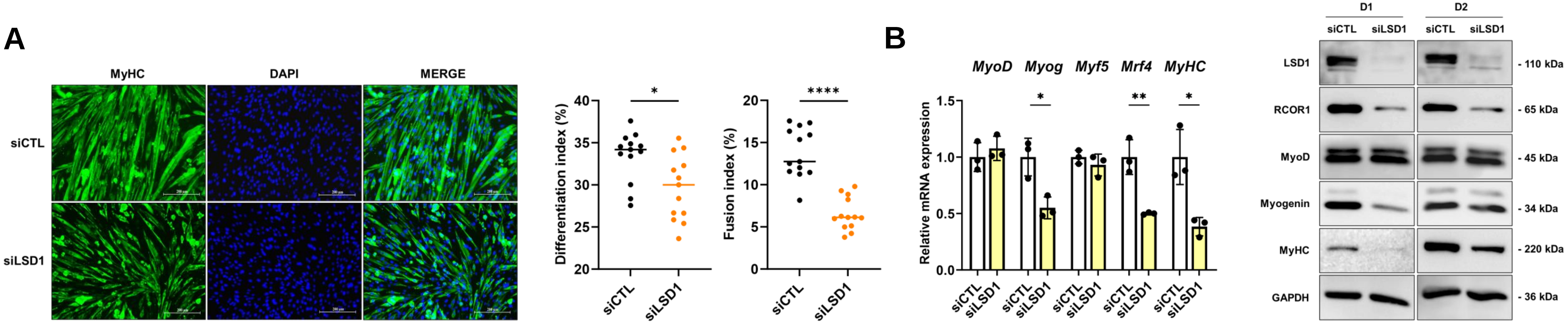

Figure S4. Primary myoblasts isolated from mouse limb muscles were transfected with siCTL or siLSD1 and induced to differentiate for 2 days. (**A**) Terminally differentiated myotubes were visualized by anti-MyHC immunofluorescent staining (green, MyHC; blue, DAPI) at day 2 in DM. The differentiation index and fusion index were counted. Scale bar 200 µm. (**B**) qPCR and immunoblotting were performed to detect the mRNA and protein levels of RCOR1, LSD1, MyoD, Myogenin, Myf5 and Mfr4 at day 1 and MyHC at day 2 in DM. Pictures are representatives of 3 independent experiments. Data are presented as mean ± SD. *P < 0.05; **P< 0.01; ****P< 0.0001. Student's t-test was performed.

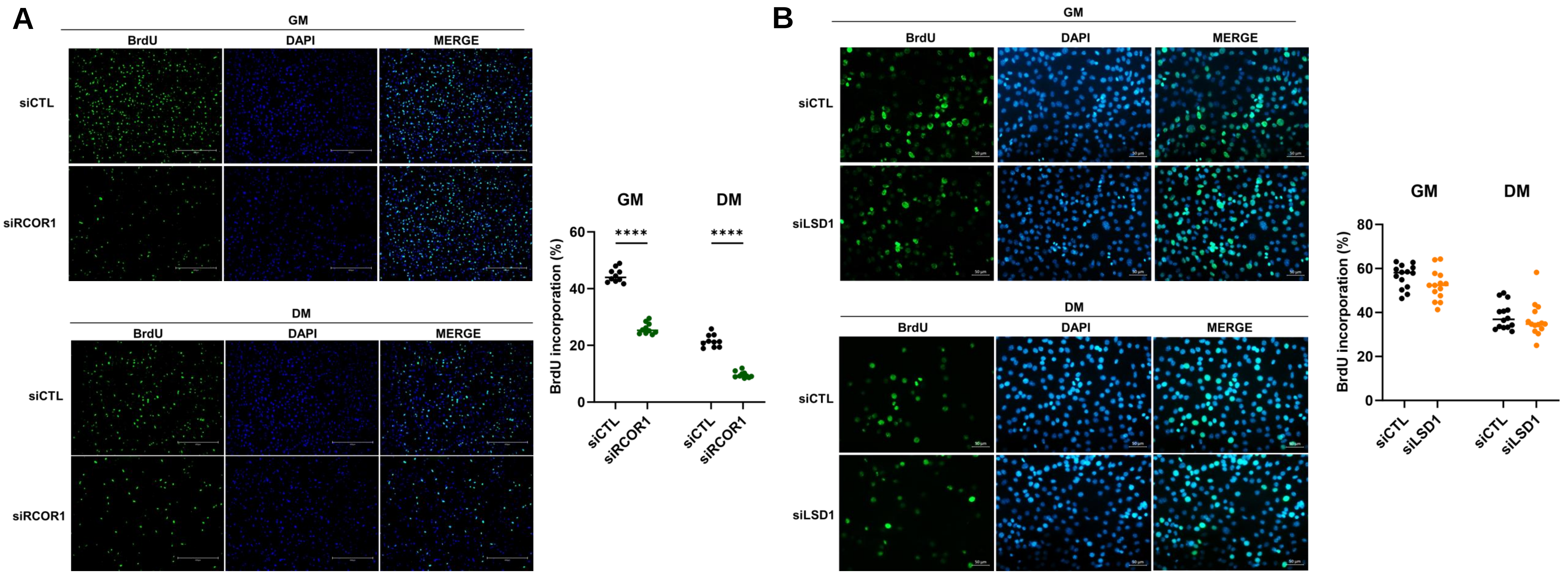

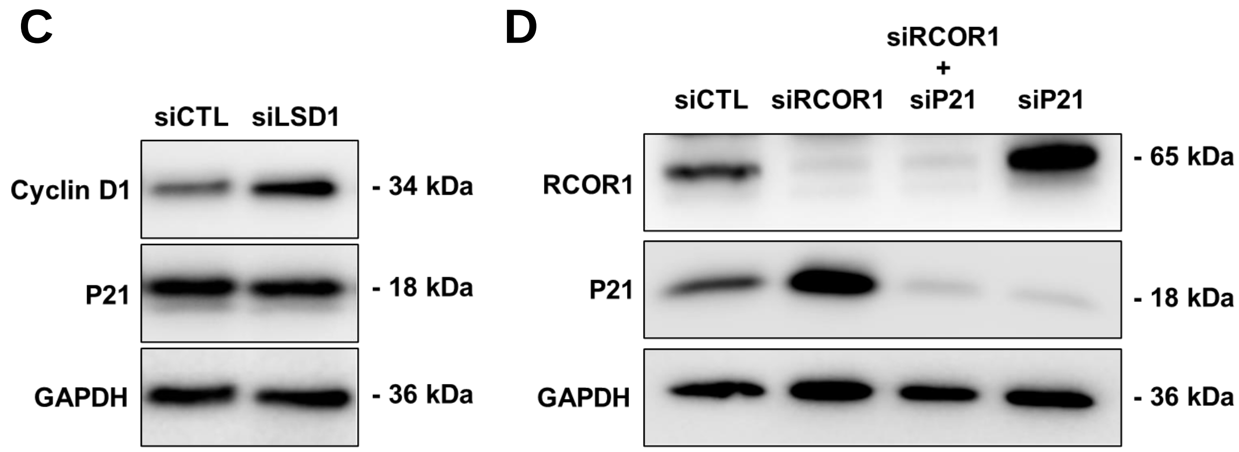

Figure S5. (**A**) Primary mouse myoblasts transfected with siCTL or siRCOR1 were cultured in GM and then switched into fresh GM or DM for additional 1 day. After 1 day, cells were stimulated with 10 µM BrdU for 4 hours and then immunostained for the detection of BrdU incorporation (green BrdU; blue DAPI). The percentage of the number of BrdU positive cells was calculated. Scale bar 450 µm. (**B**) C2C12 transfected with siCTL or siLSD1 were cultured in GM and then switched into fresh GM or DM for additional 1 day. After 1 day, cells were stimulated with 10 µM BrdU for 4 hours and then immunostained for the detection of BrdU incorporation (green BrdU; blue DAPI). The percentage of the number of BrdU positive cells was calculated. Scale bar 50 µm. Transfected C2C12 cells cultured in DM for 1 day were analysed for protein levels of (**C**) cell cycle regulators P21 and Cyclin D1 and (**D**) P21 and RCOR1 by immunoblotting. Pictures are representatives of 3 independent experiments. Data are presented as means ± SD. ****P< 0.0001. Student's t-test was performed.

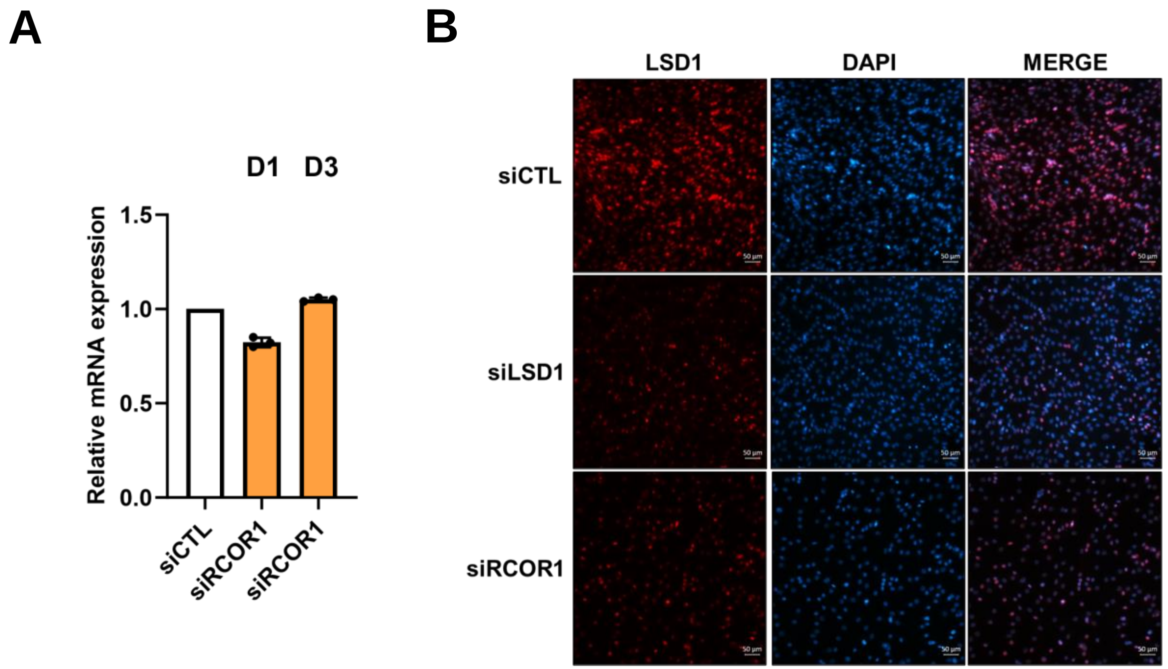

Figure S6. (**A**) C2C12 cells were transfected with siCTL and siRCOR1 and cells were analysed for *Lsd1* mRNA expression by qPCR during 3-day differentiation. (**B**) C2C12 were transfected with siCTL, siLSD1 and siRCOR1 and induced to differentiate for 3 days. LSD1 was detected by immunofluorescence staining (red, LSD1; blue, DAPI). Scale bar 50 µm. Pictures are representatives of 3 independent experiments. Data are presented as means ± SD. One-way ANOVA was performed.

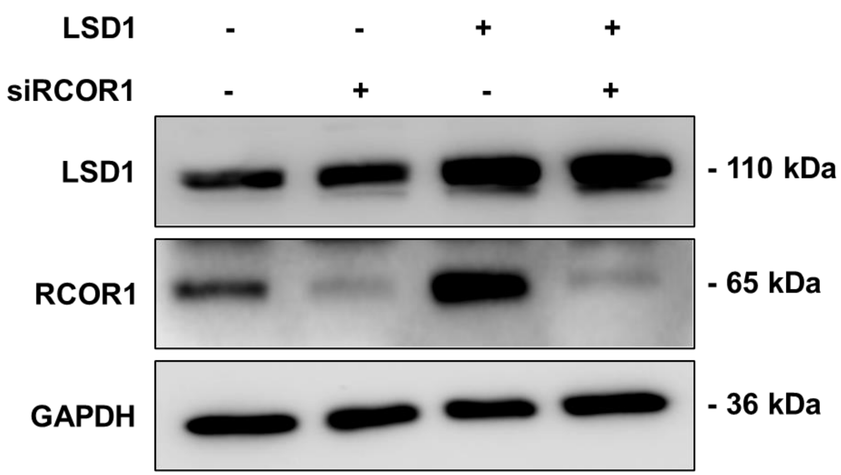

Figure S7. C2C12 cells were transfected with pCMV3-Flag-mKDM1A plasmid or respective empty vector for 24 hours, and subsequently with control siRNA or RCOR1 siRNA for additional 24 hours. Cells were then switched into fresh DM for additional 1 day. LSD1 and RCOR1 protein levels were determined by immunoblotting. Representative blot from 3 independent experiments is shown.
