# Supplementary material for: RCOR1 promotes myoblast differentiation and muscle regeneration
Source: Cell Death Discov. 2025 Jul 1;11:298. doi: 10.1038/s41420-025-02568-9 (PMC12217761; doi:10.1038/s41420-025-02568-9)

Figure 1

B

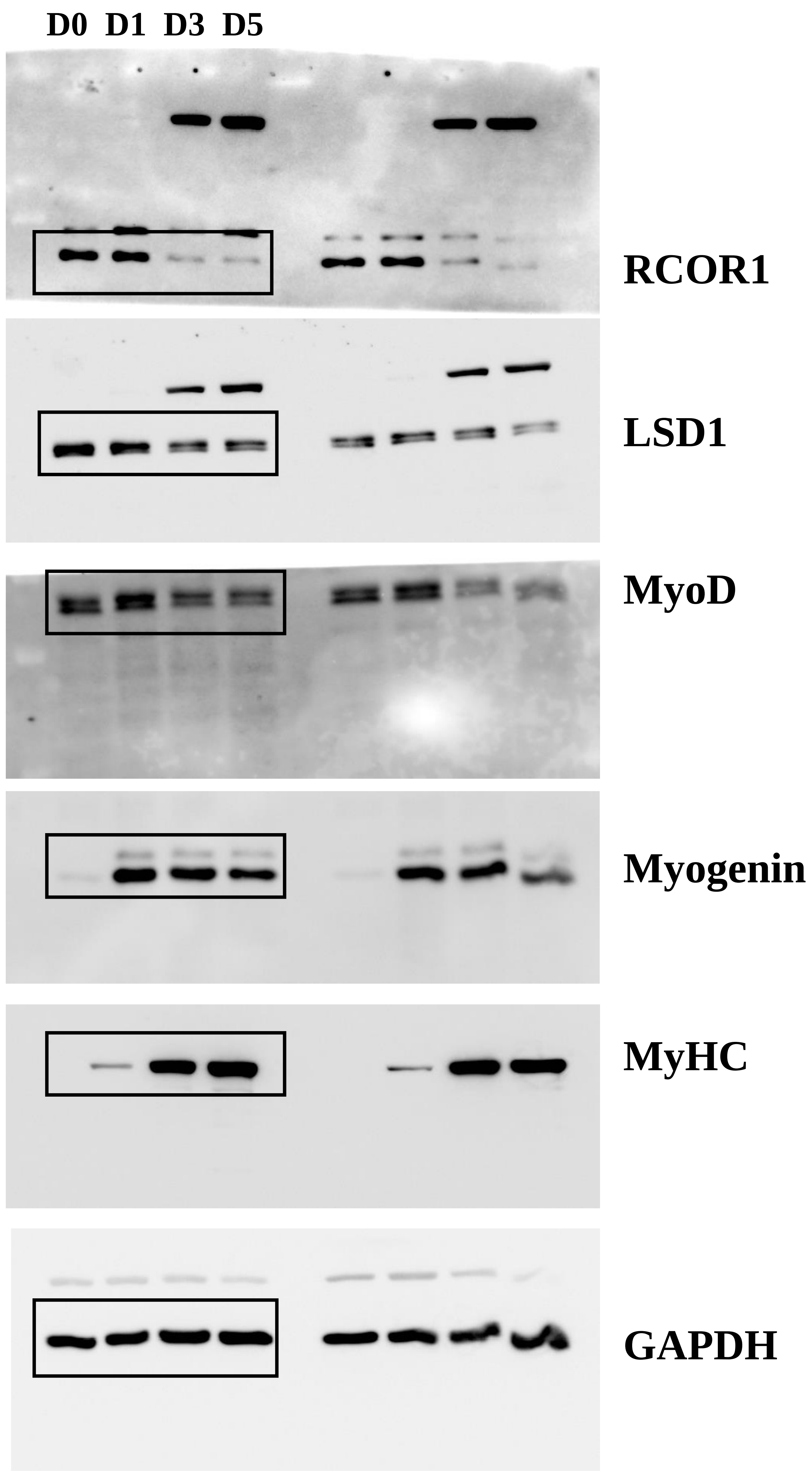
D0 D1 D3 D5
RCOR1
LSD1
MyoD
Myogenin
MyHC
GAPDH

D

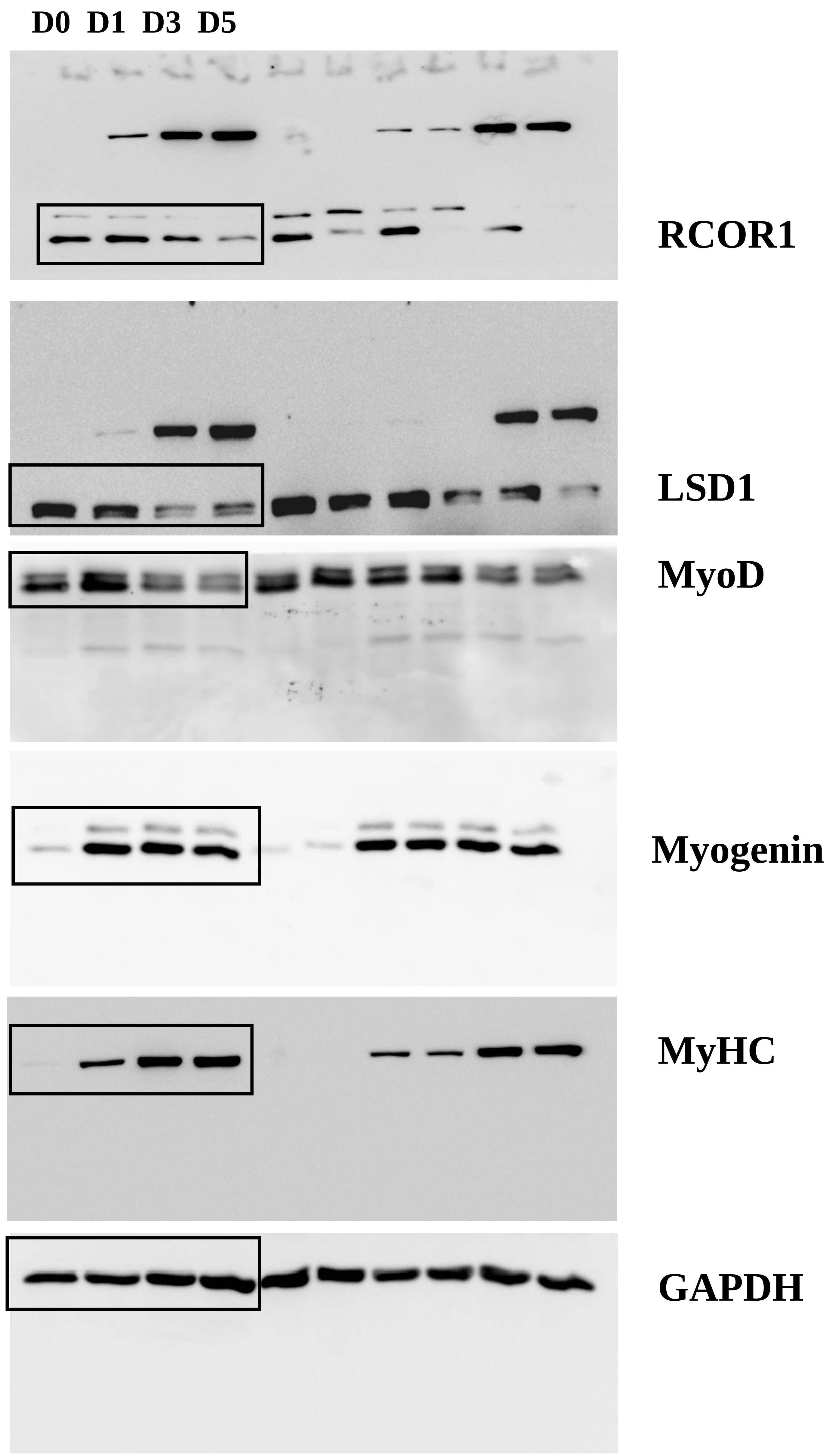
D0 D1 D3 D5
RCOR1
LSD1
MyoD
Myogenin
MyHC
GAPDH

Figure 2

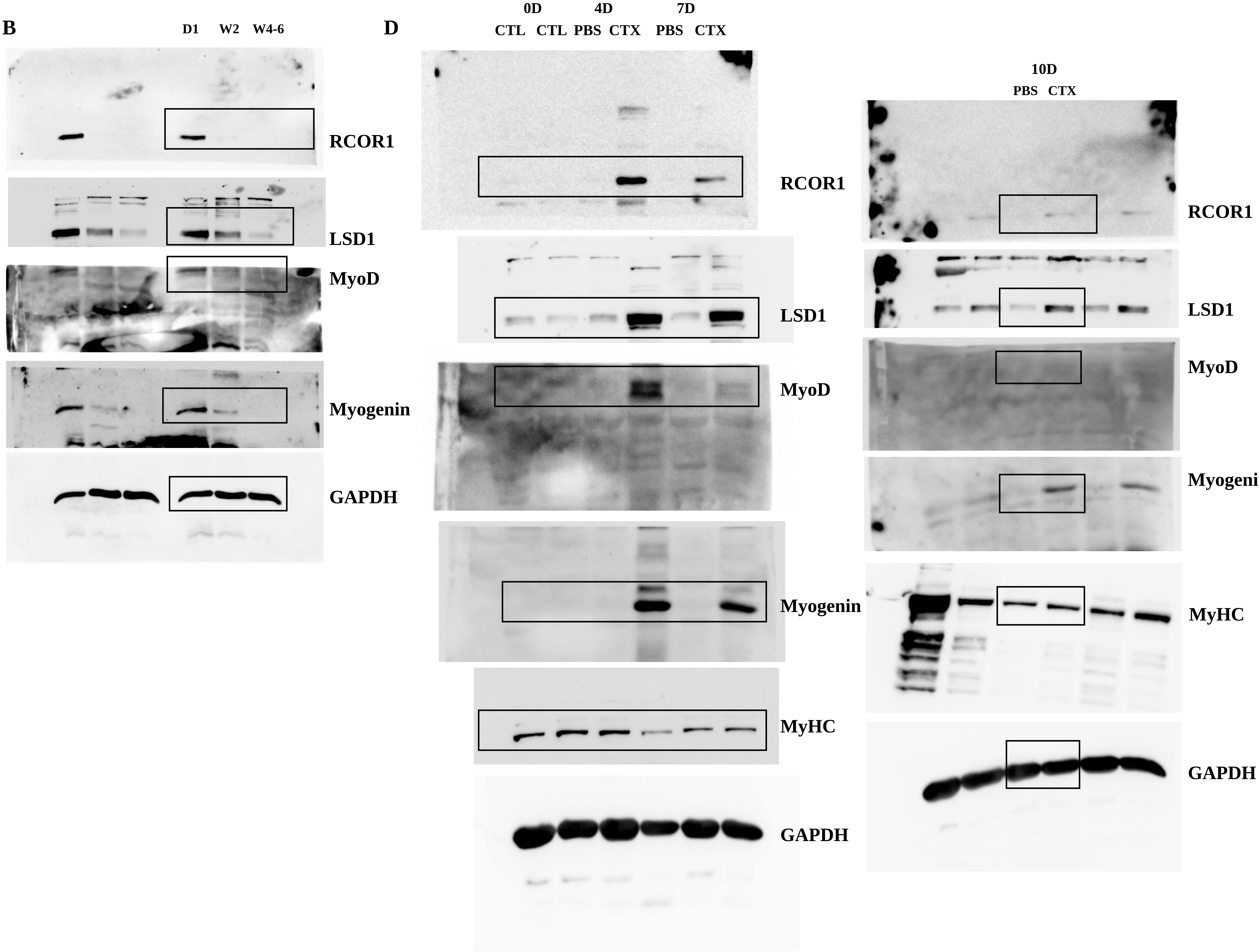
B
D1
W2
W4-6
RCOR1
LSD1
MyoD
Myogenin
GAPDH
D
0D
4D
7D
CTL
CTL
PBS
CTX
PBS
CTX
RCOR1
LSD1
MyoD
Myogenin
MyHC
GAPDH
10D
PBS
CTX
RCOR1
LSD1
MyoD
Myogeni
MyHC
GAPDH

Figure 3

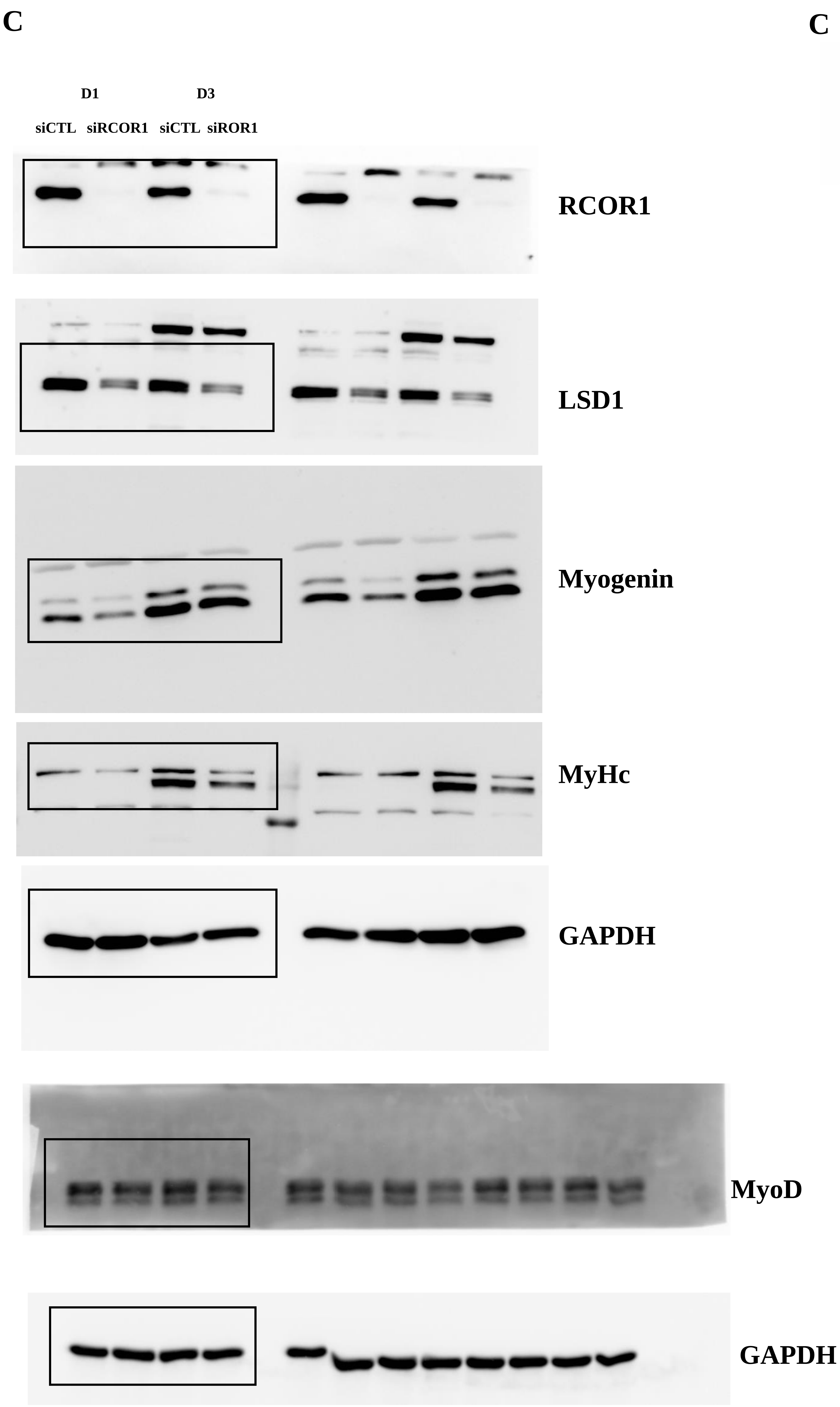

Figure 4

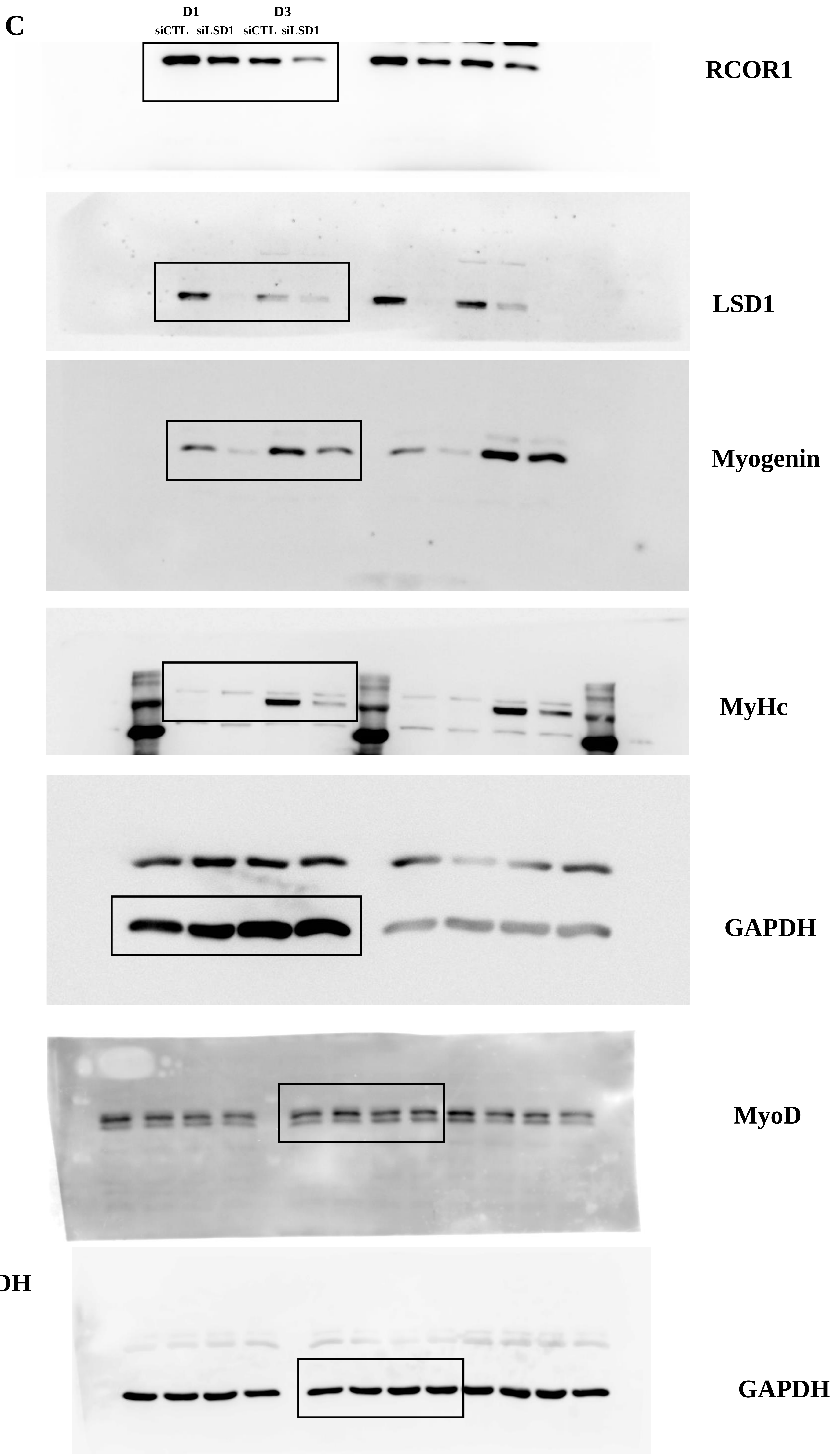

Figure 5

E

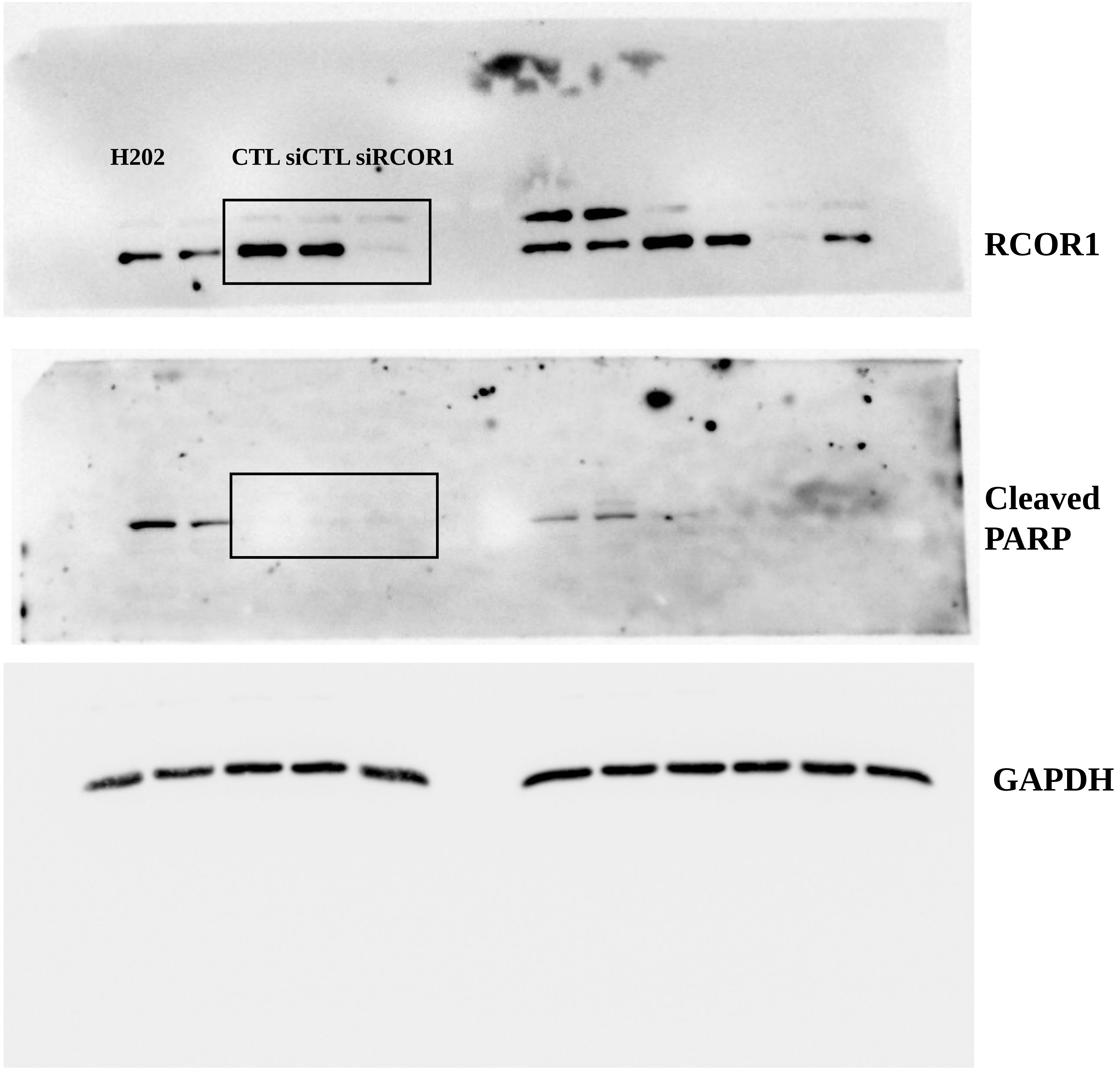

Figure 6

B

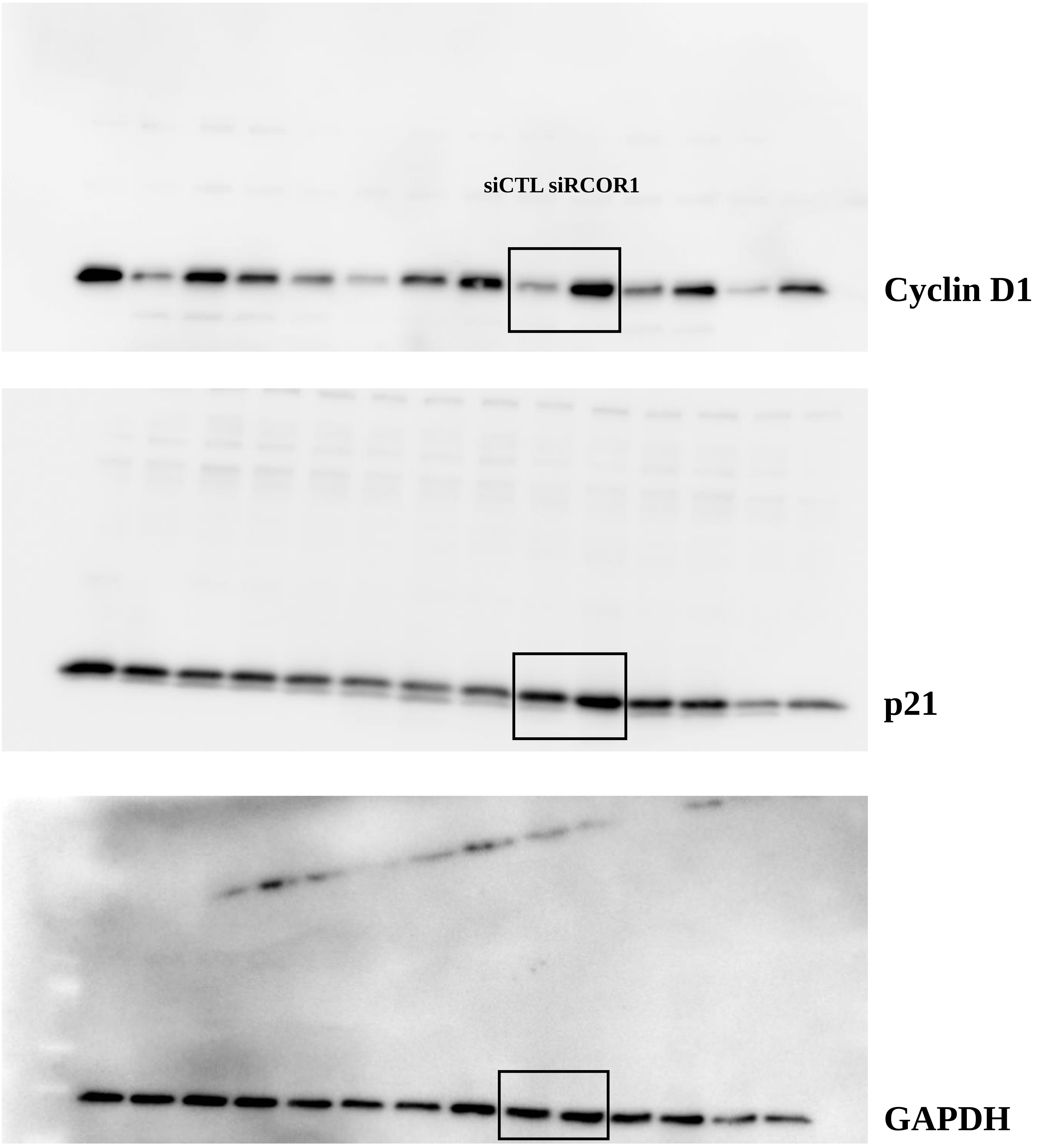

Figure 6

E

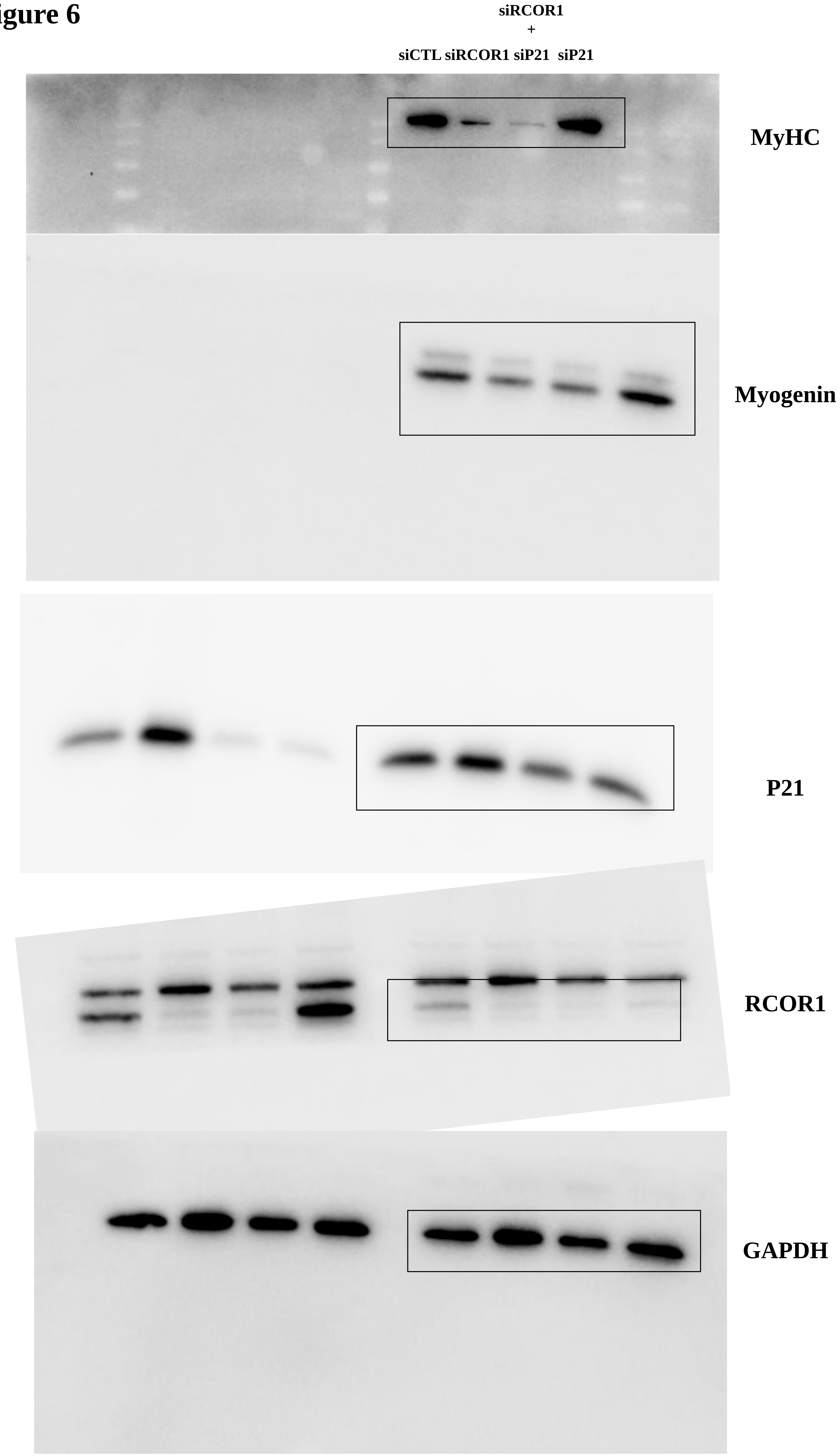

siRCOR1
+
siCTL siRCOR1 siP21 siP21
MyHC
Myogenin
P21
RCOR1
GAPDH

Figure 7

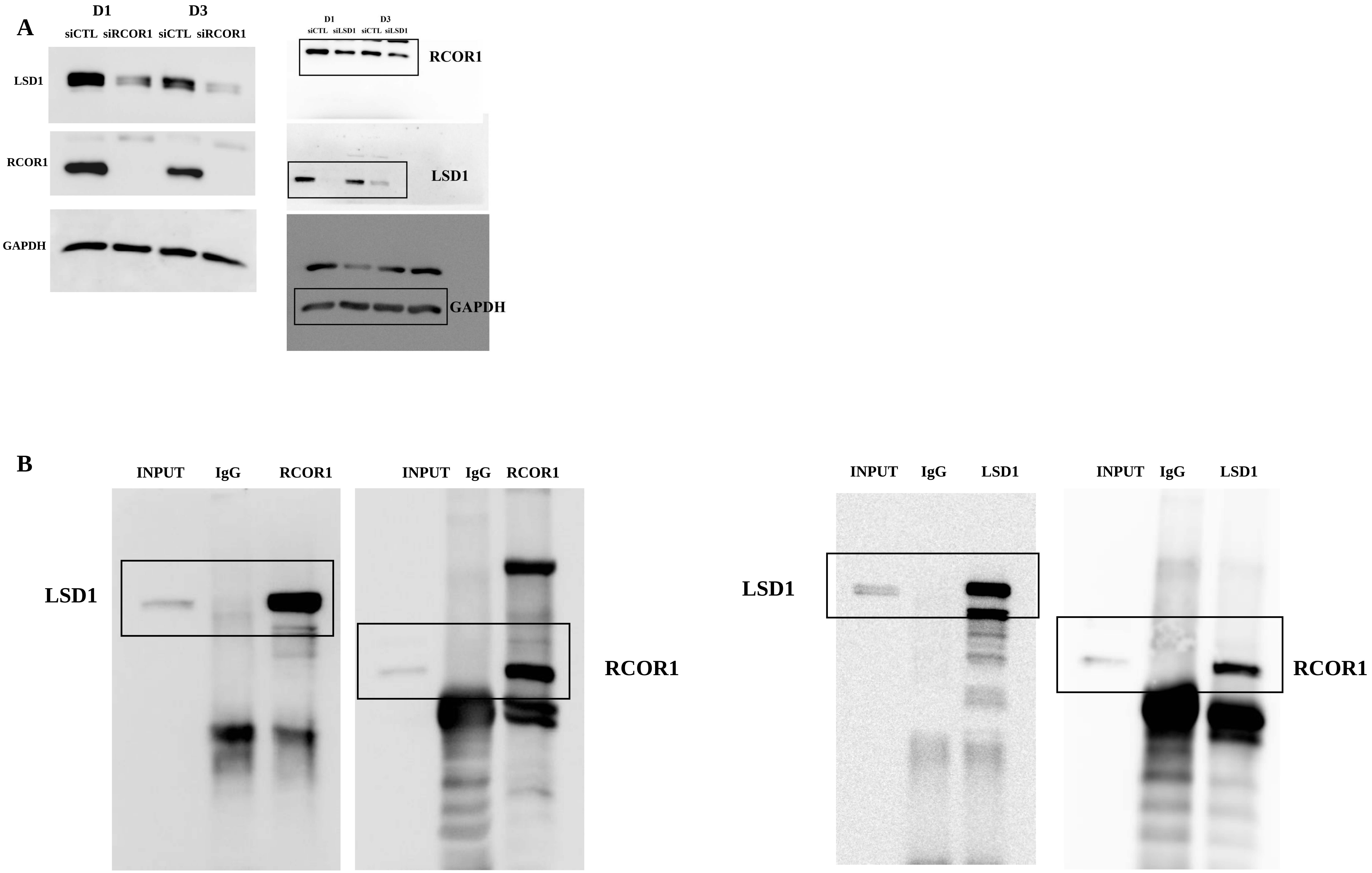

A
D1
D3
siCTL
siRCOR1
siCTL
siRCOR1
LSD1
RCOR1
GAPDH
D1
D3
siCTL
siLSD1
siCTL
siLSD1
RCOR1
LSD1
GAPDH
B
INPUT
IgG
RCOR1
INPUT
IgG
RCOR1
LSD1
RCOR1
INPUT
IgG
LSD1
INPUT
IgG
LSD1
LSD1
RCOR1

Figure 7

C

siCTL siRCOR1

0 2 4 6 0 2 4 6 6 (MG132)

LSD1

RCOR1

GAPDH

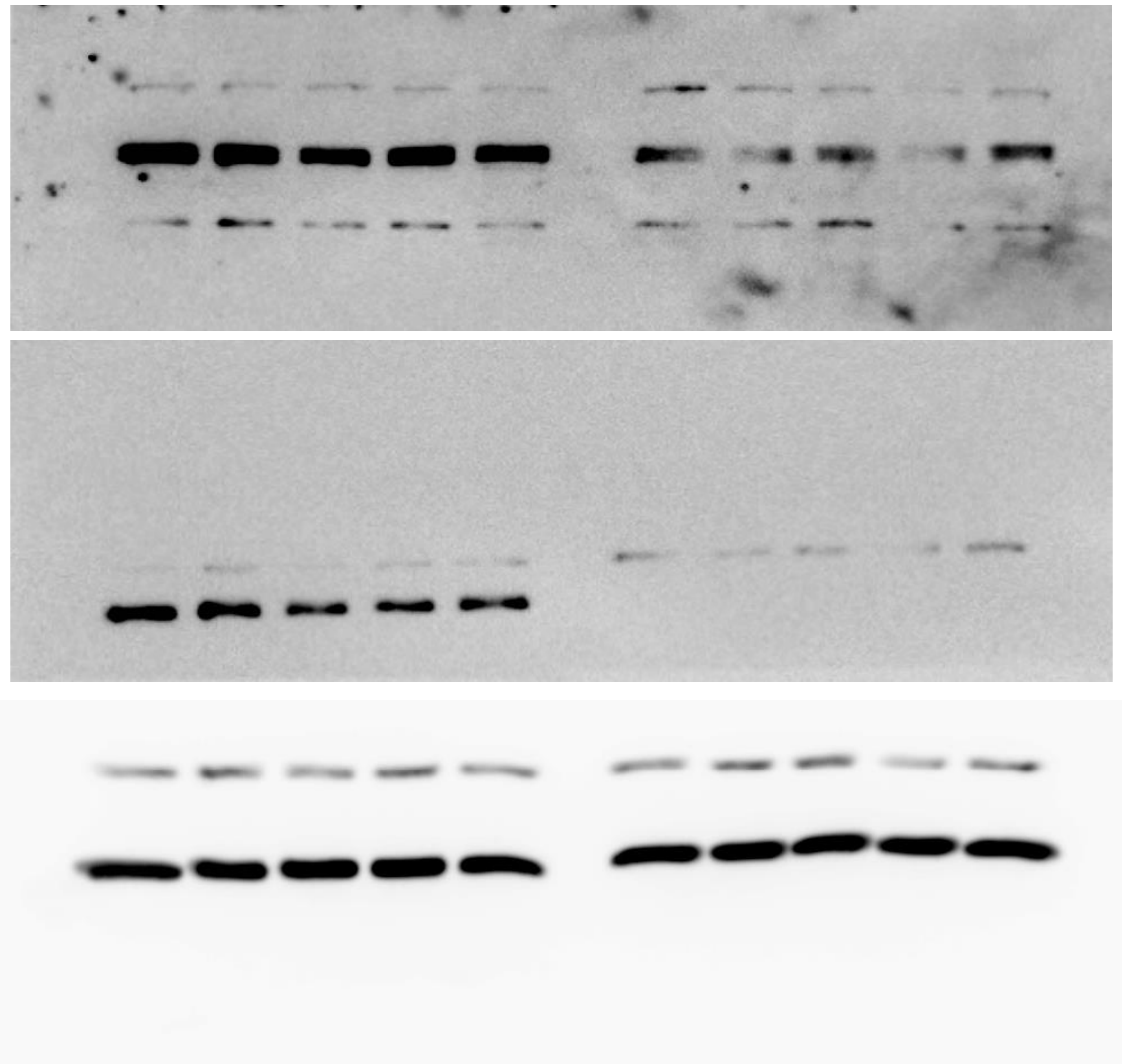

D

INPUT IP LSD1

siCTL siRCOR1 IgG siCTL siRCOR1 IgG

MG132 - + - + MG132 - + - +

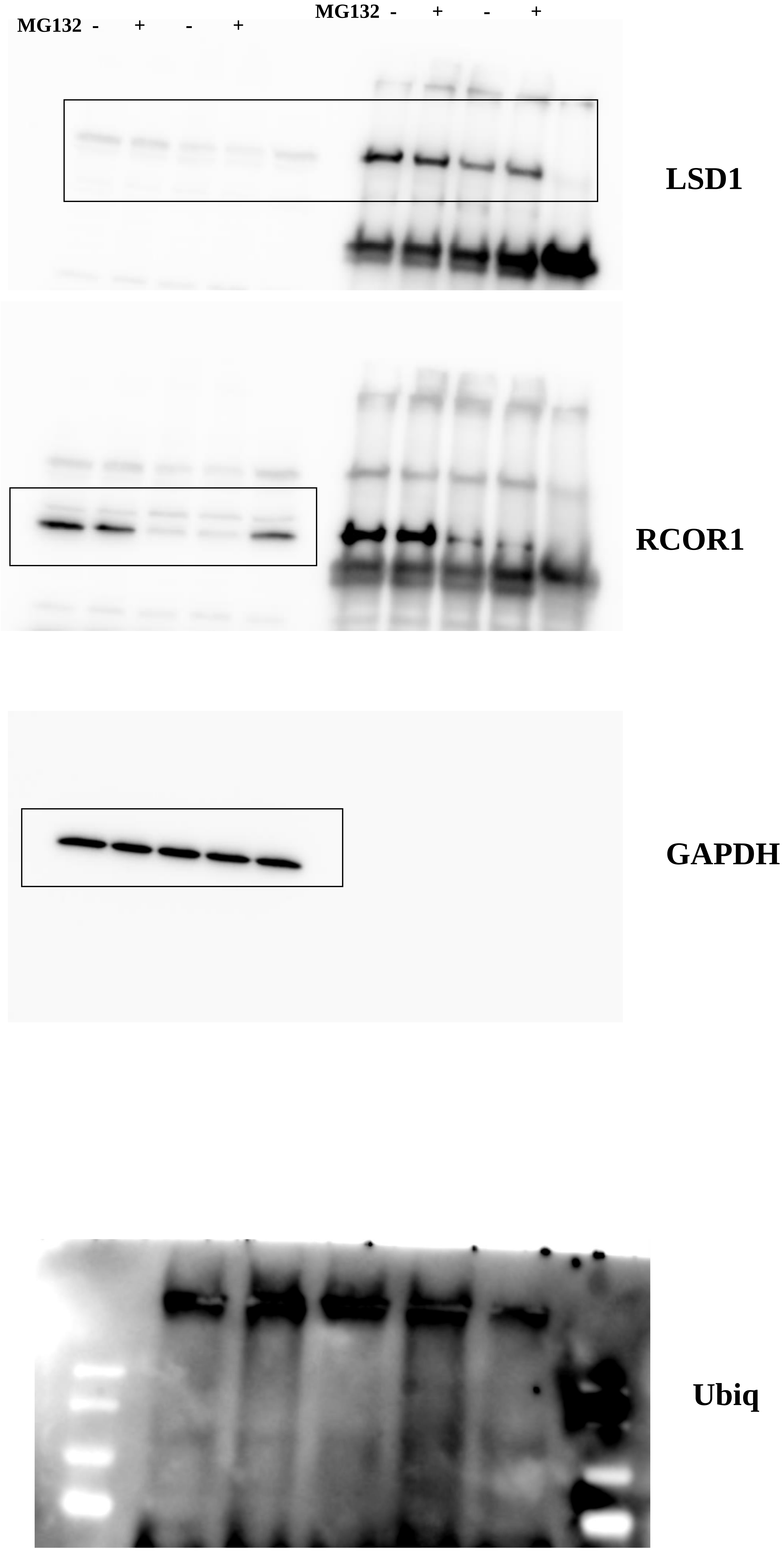

LSD1

RCOR1

GAPDH

IP LSD1

Ubiq

Figure 7

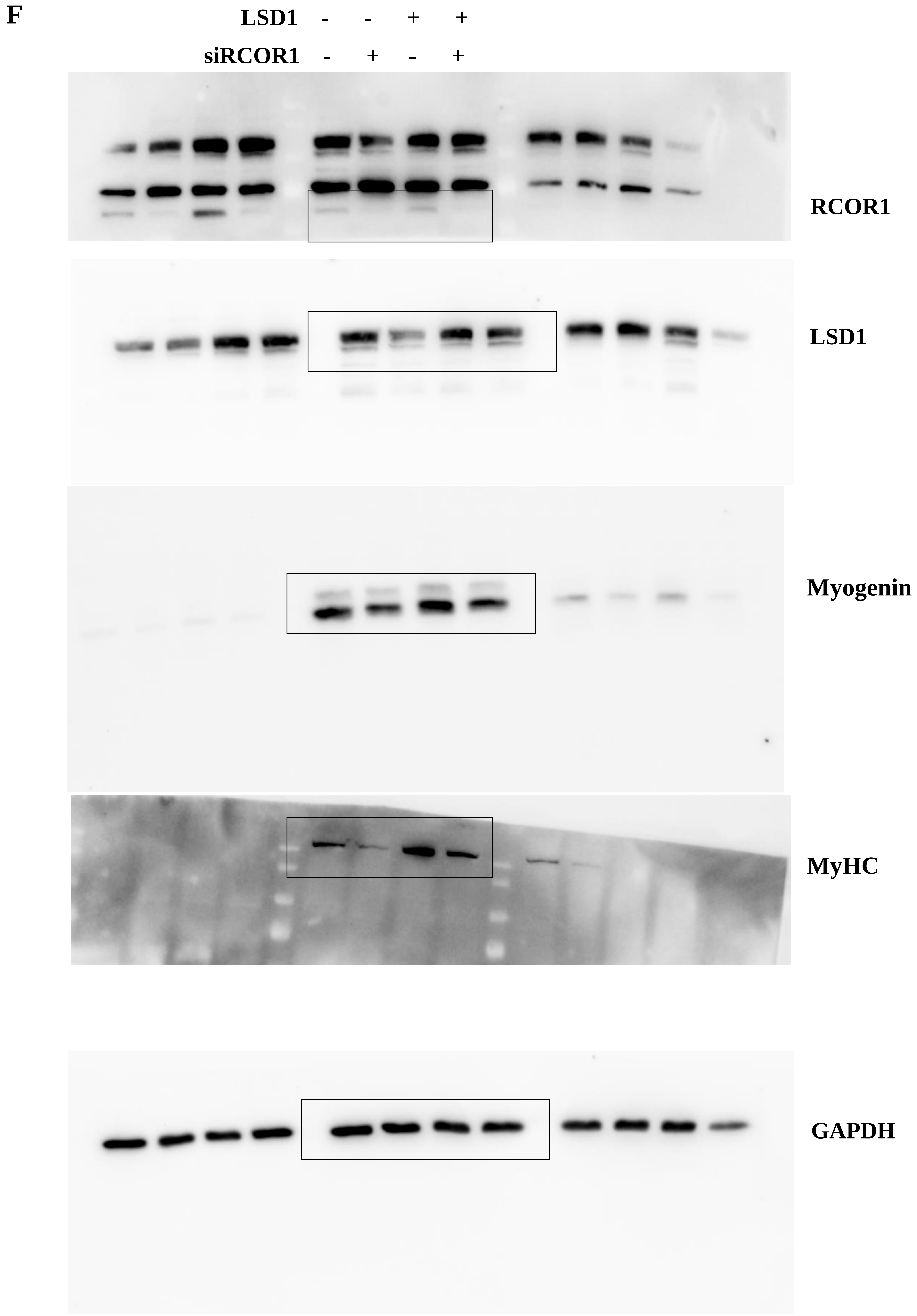

Figure 7

G

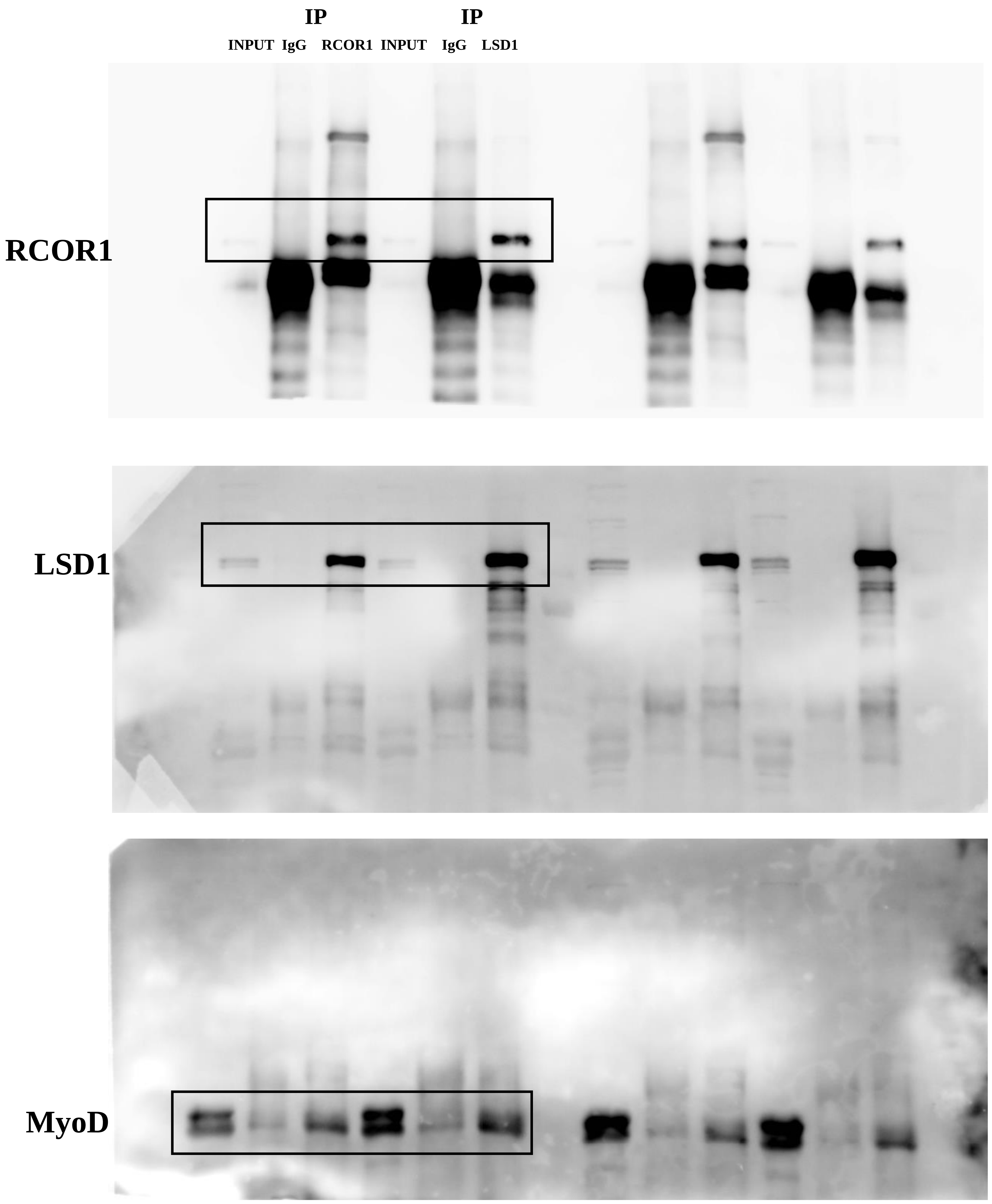

Figure 8

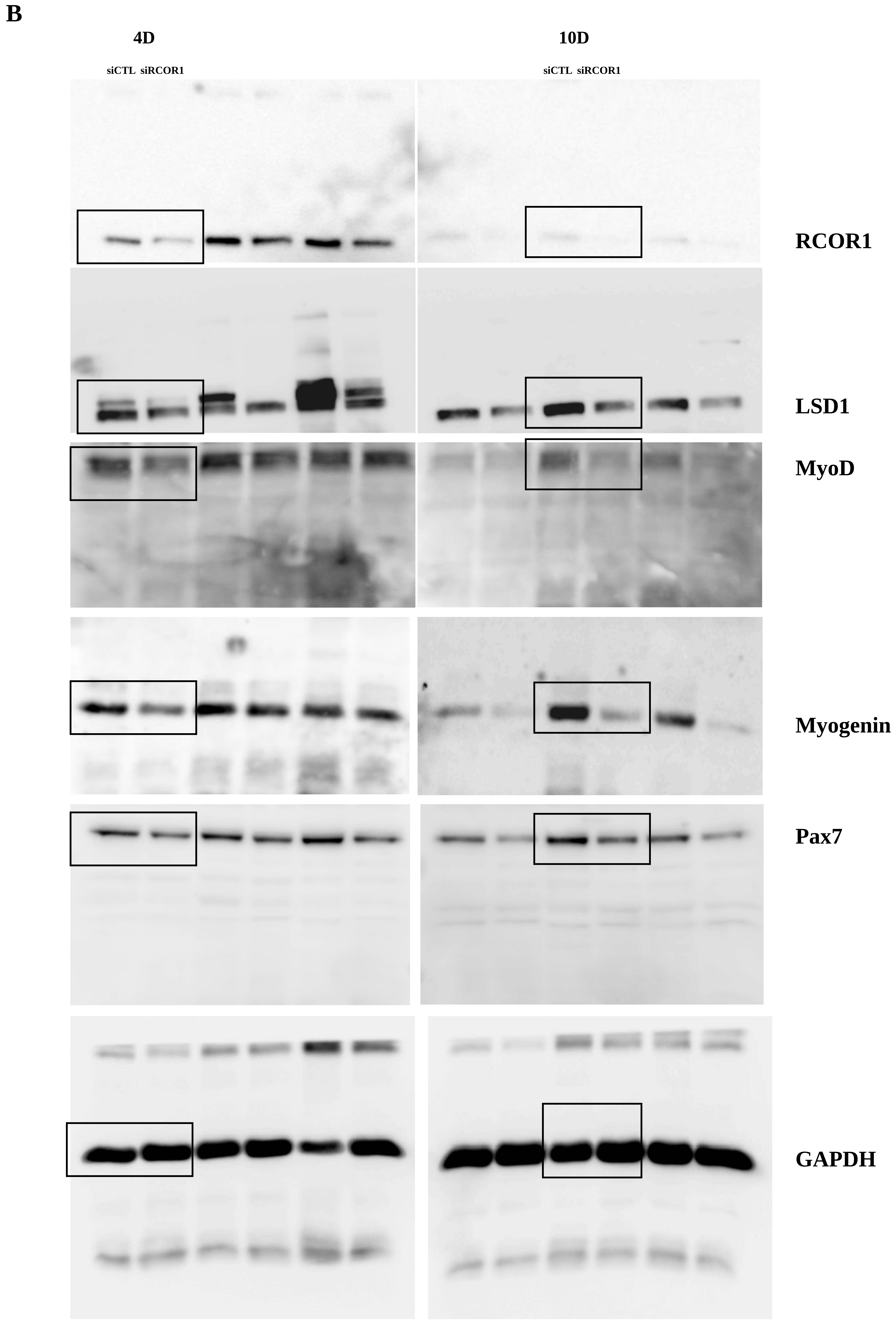
B
4D
10D
siCTL siRCOR1
siCTL siRCOR1
RCOR1
LSD1
MyoD
Myogenin
Pax7
GAPDH

Figure S3

A

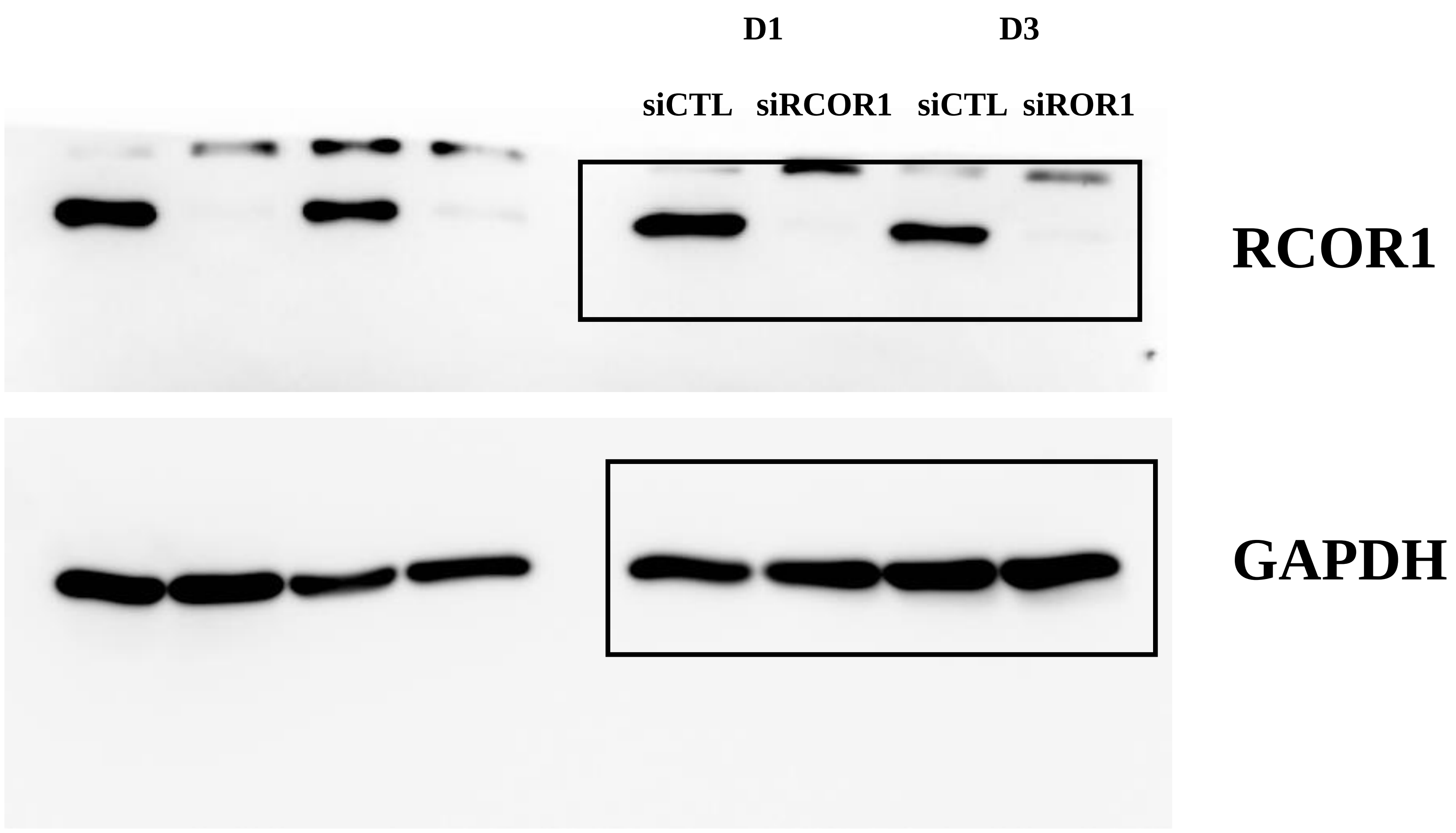

Figure S3

C

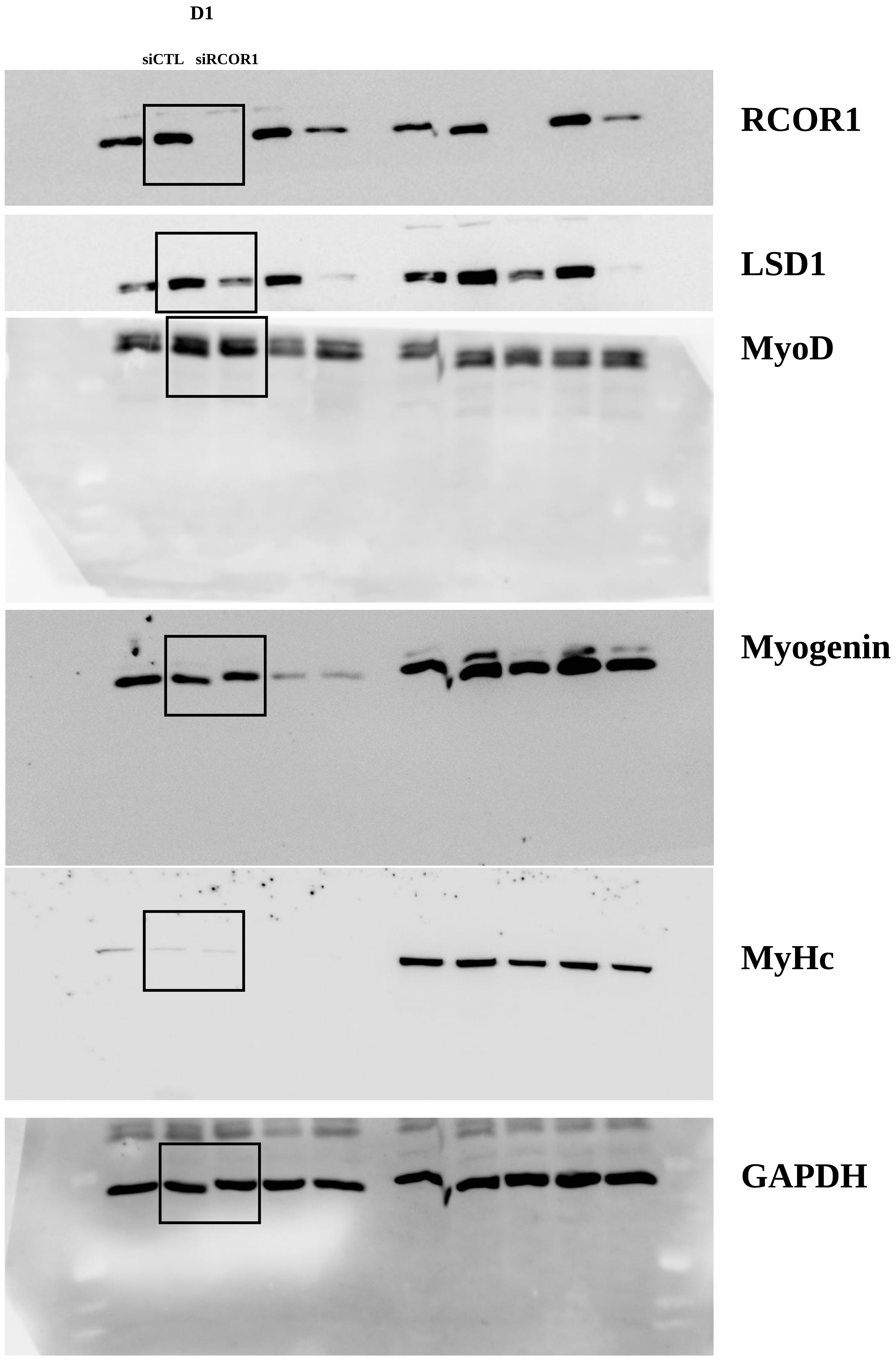
D1
siCTL
siRCOR1
RCOR1
LSD1
MyoD
Myogenin
MyHc
GAPDH

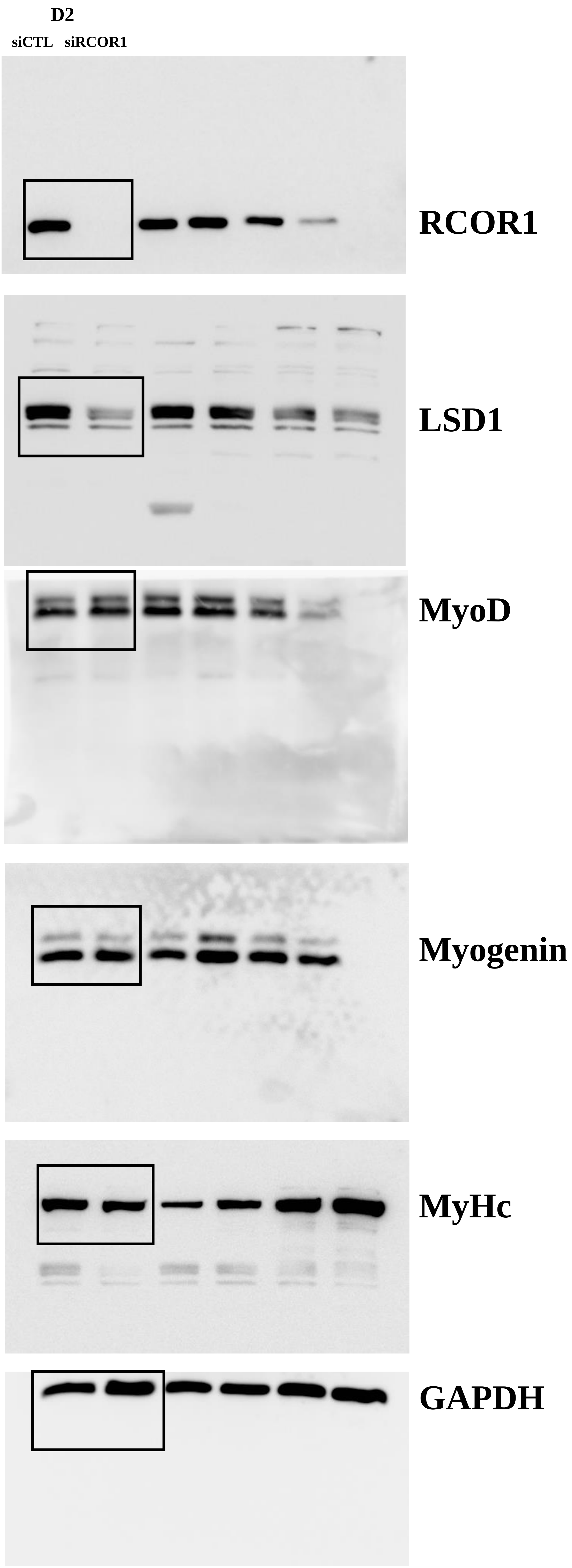
D2
siCTL
siRCOR1
RCOR1
LSD1
MyoD
Myogenin
MyHc
GAPDH

Figure S4

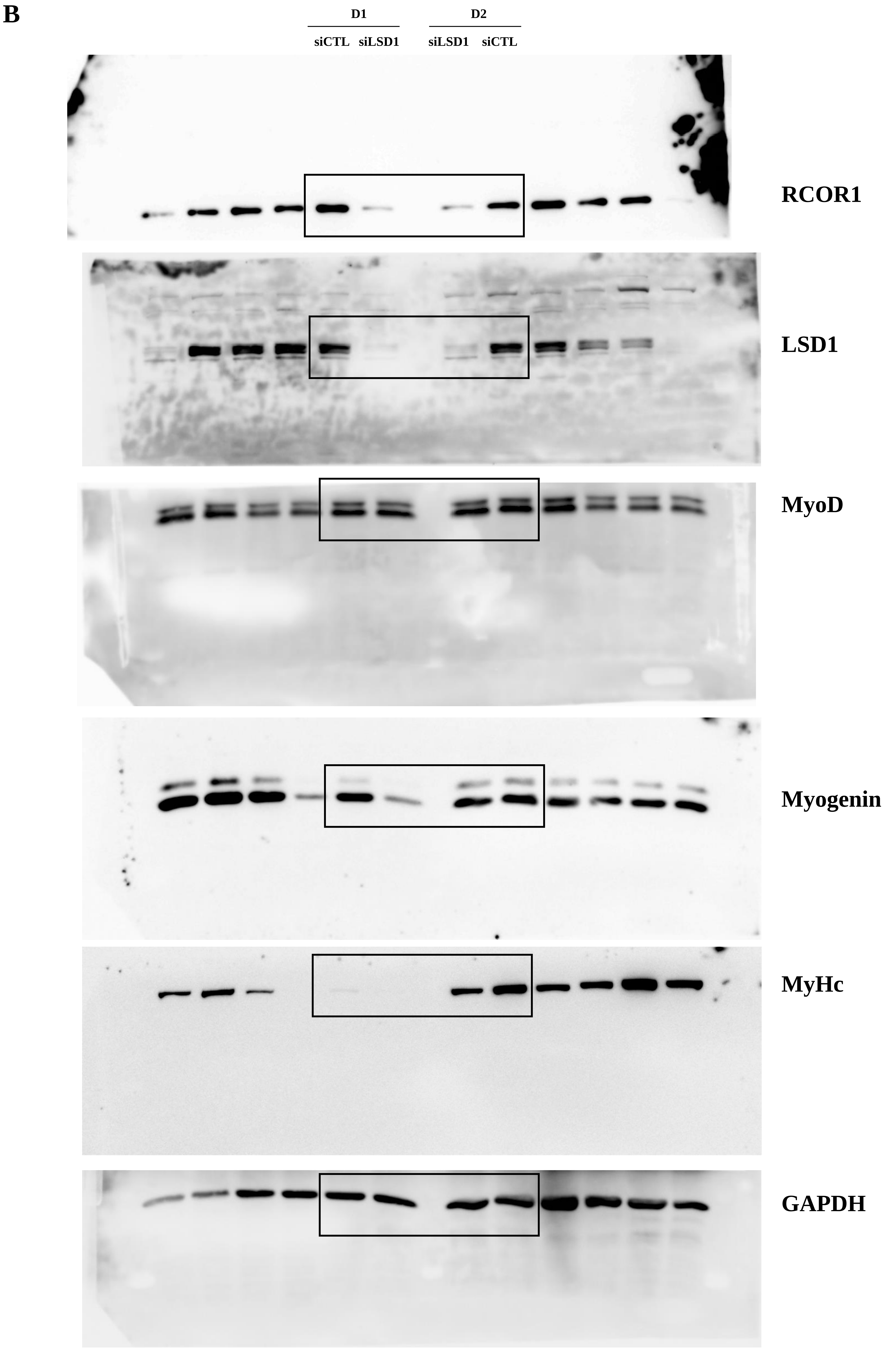
B
D1
D2
siCTL
siLSD1
siLSD1
siCTL
RCOR1
LSD1
MyoD
Myogenin
MyHc
GAPDH

Figure S5

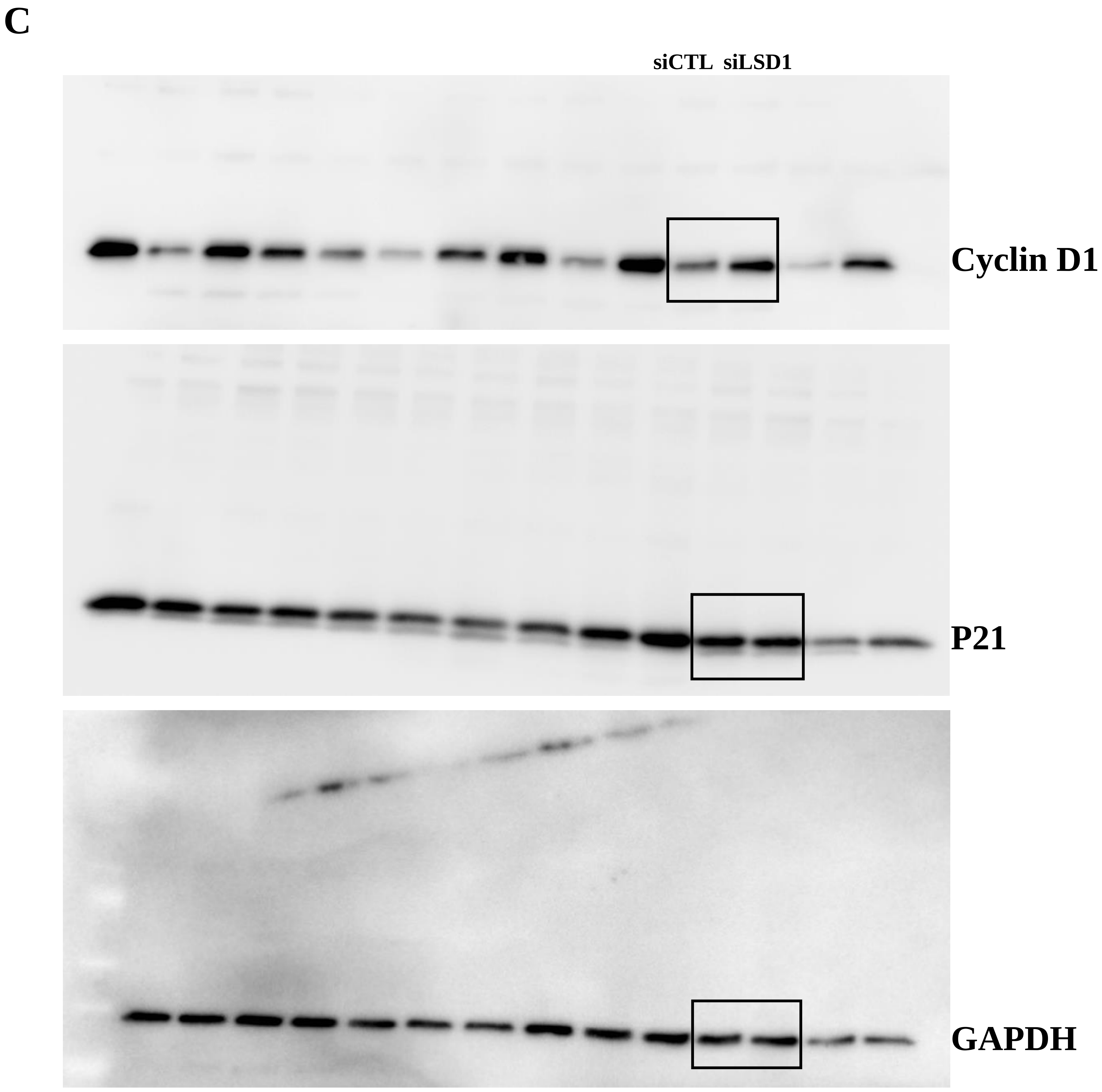
C
siCTL
siLSD1
Cyclin D1
P21
GAPDH

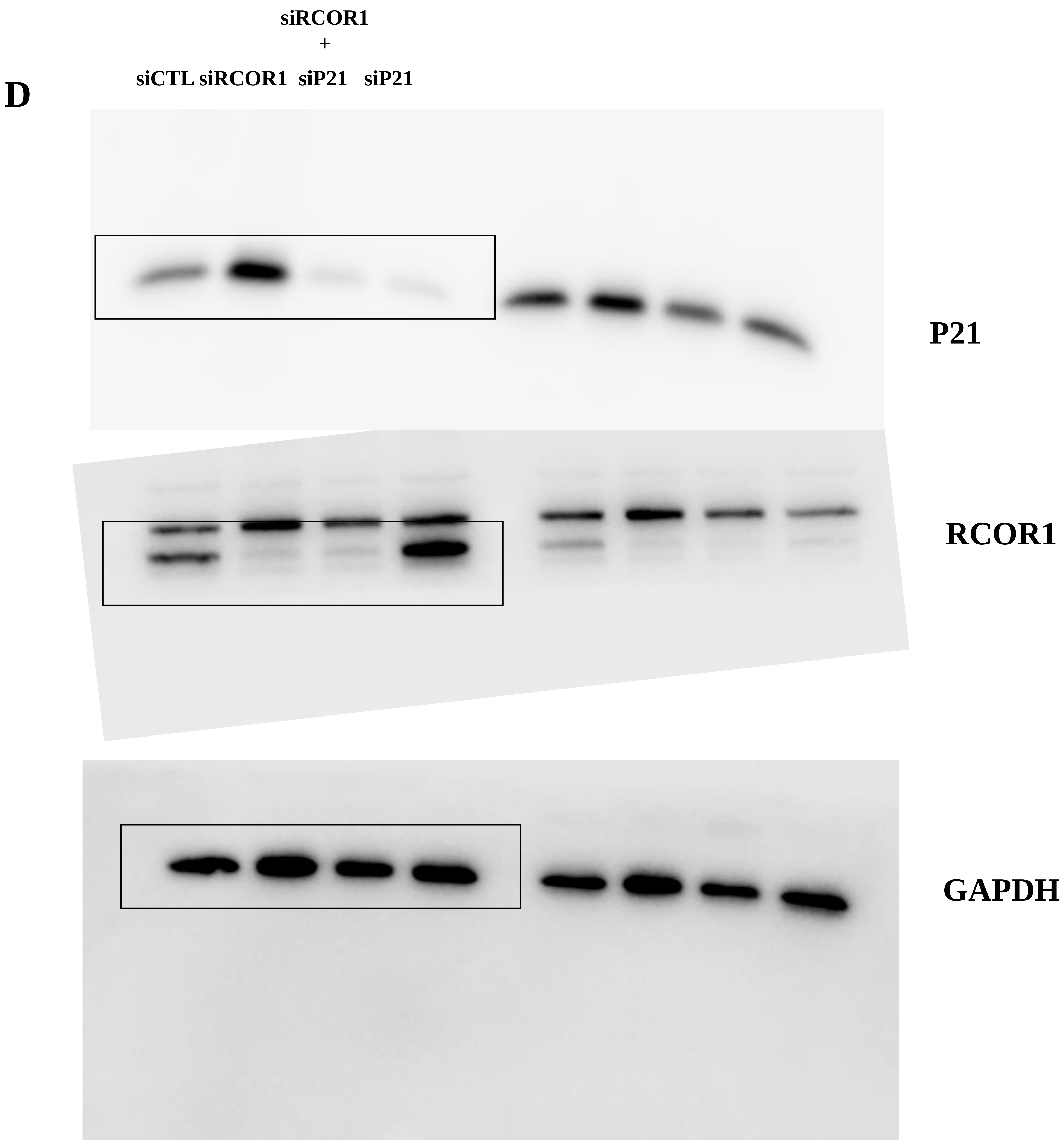
D
siRCOR1
+
siCTL
siRCOR1
siP21
siP21
P21
RCOR1
GAPDH

Figure S7

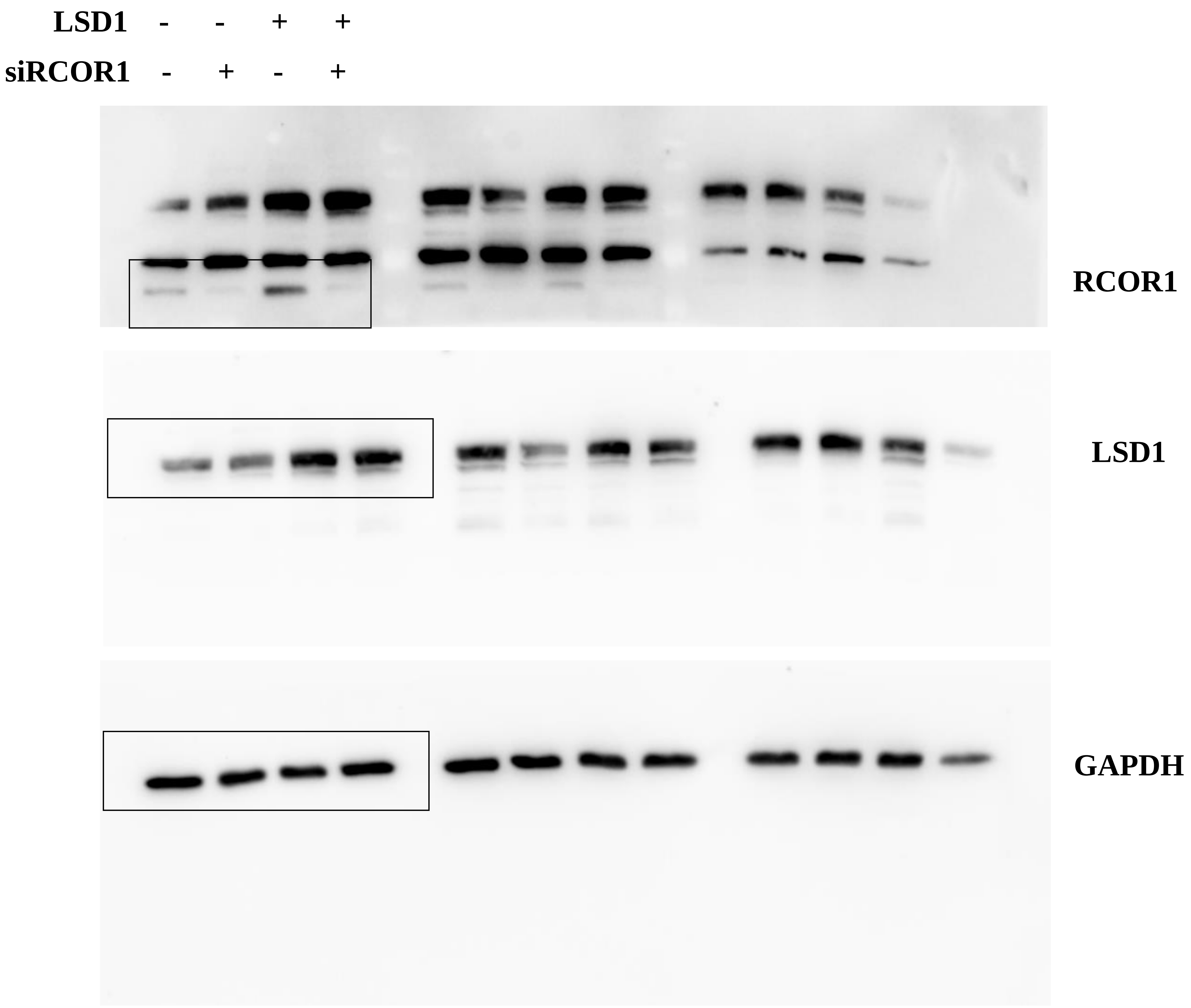

Supplement: Supplementary file 4 — Original immunoblots [file 41420_2025_2568_MOESM4_ESM.pdf]
